# Determination and analysis of agonist and antagonist potential of naturally occurring flavonoids for estrogen receptor (ERα) by various parameters and molecular modelling approach

**DOI:** 10.1038/s41598-019-43768-5

**Published:** 2019-05-15

**Authors:** Ninad V. Puranik, Pratibha Srivastava, Gaurav Bhatt, Dixcy Jaba Sheeba John Mary, Anil M. Limaye, Jayanthi Sivaraman

**Affiliations:** 1 Bioprospecting Group, Agharkar Research Institute, G. G, Agarkar Road, Pune, 411004 Maharashtra India; 2 Department of Biosciences and Bioengineering, Indian Institute of Technology, Guwahati, 781039 Assam India; 3 Savitribai Phule Pune University, Ganeshkhind, Pune, 411007 India; 4 Computational Drug Design Lab, School of Bio Sciences and Technology, Vellore Institute of Technology, Vellore, 632014 Tamil Nadu India

**Keywords:** Virtual drug screening, Screening

## Abstract

Most estrogen receptor α (ERα) ligands target the ligand binding domain (LBD). Agonist 17β-estradiol (E_2_) and tamoxifen (TM, known SERM), bind to the same site within the LBD. However, structures of ligand-bound complexes show that E_2_ and TM induce different conformations of helix 12 (H12). During the molecular modelling studies of some naturally occurring flavonoids such as quercetin, luteolin, myricetin, kaempferol, naringin, hesperidin, galangin, baicalein and epicatechin with human ERα (3ERT and 1GWR), we observed that most of the ligands bound to the active site pocket of both 3ERT and 1GWR. The docking scores, interaction analyses, and conformation of H12 provided the data to support for the estrogenic or antiestrogenic potential of these flavonoids to a limited degree. Explicit molecular dynamics for 50 ns was performed to identify the stability and compatibility pattern of protein-ligand complex and RMSD were obtained. Baicalein, epicatechin, and kaempferol with 1GWR complex showed similar RMSD trend with minor deviations in the protein backbone RMSD against 1GWR-E_2_ complex that provided clear indications that ligands were stable throughout the explicit molecular simulations in the protein and outcome of naringin-3ERT complex had an upward trend but stable throughout the simulations and all molecular dynamics showed stability with less than overall 1 Å deviation throughout the simulations. To examine their estrogenic or antiestrogenic potential, we studied the effect of the flavonoids on viability, progesterone receptor expression and 3xERE/3XERRE-driven reporter gene expression in ERα positive and estrogen responsive MCF-7 breast cancer cells. Epicatechin, myricetin, and kaempferol showed estrogenic potential at 5 µM concentration.

## Introduction

Estrogens cohere with the estrogen receptors (ERs) and employ their physiological effects. They are the members of the nuclear receptor (NR) superfamily^1,2^ of the ligand-activated transcription factors. The ERα plays a vital role in the delineation and maintenance of neural, skeletal, cardiovascular, and reproductive tissues^3–6^. Currently, compounds which effectively modify ERα transcriptional activity are found beneficial in the treatment of osteoporosis, cardiovascular disease, and breast cancer^7^.

The ligand binding domain (LBD) of ERα is very conserved and is responsible for the ligand binding. The LBD distinguishes various molecules based on their chemical structures and properties. Endogenous estrogen E_2_ and nonsteroidal synthetic diethylstilbestrol (DES) are estrogenic ligands, whereas TM and raloxifene (RAL) are selective estrogen receptor modulators (SERMs)^8^.

**Figure 1:**
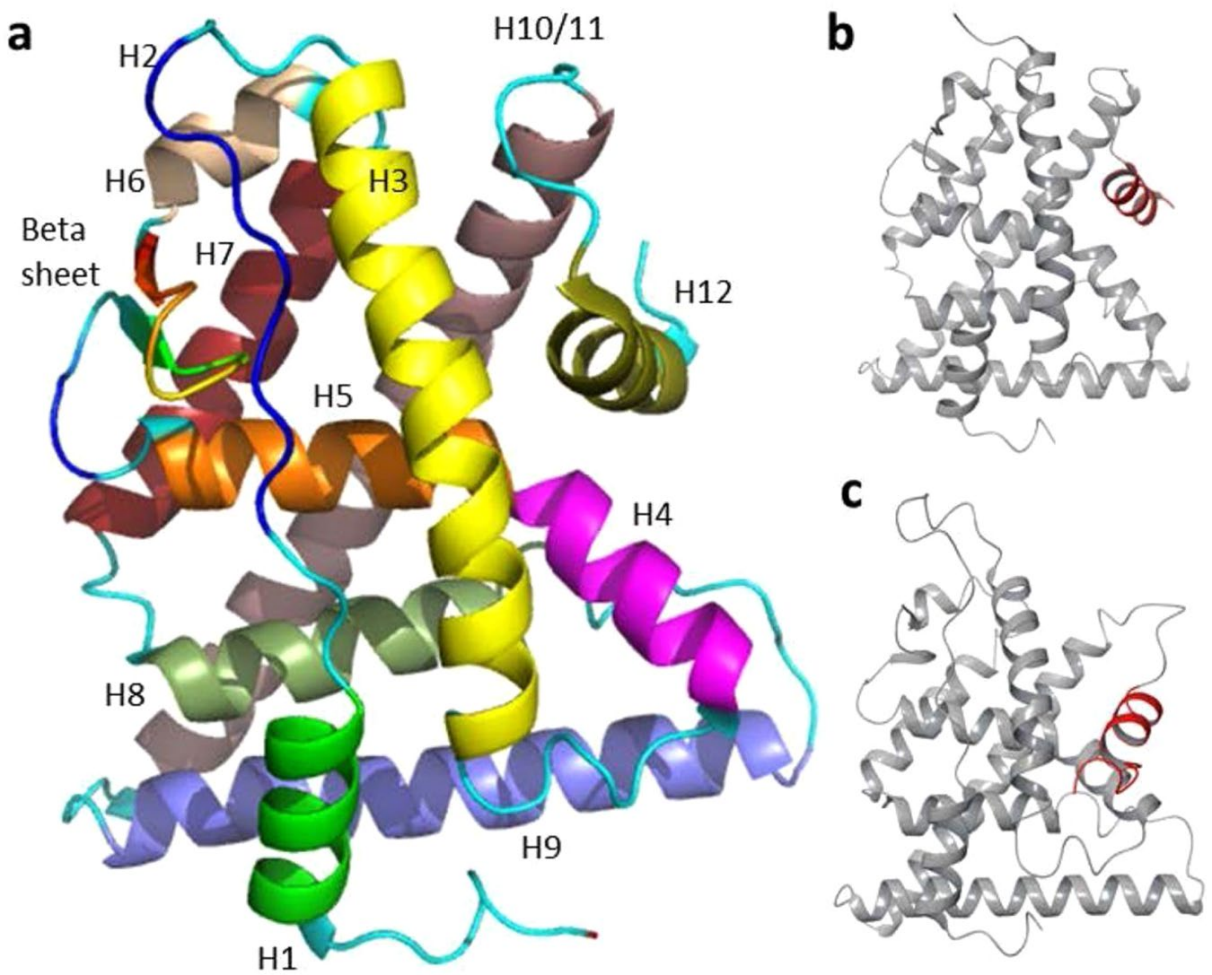
Estrogen receptor’s (ERα) ligand binding domain (LBD). (**a**) The LBD consists of twelve α-helices and a beta sheet/hairpin: The twelve α helices (H1-12) are colored differently for better distinguishability; (**b**) conformation of an active ER and (**c**) conformation of an inactive ER. The significant difference between (**b** and **c**) lies in the H12 conformation, displayed in red color.

Two distinct activation functions (AFs), which are AF-1 in the N-terminus, and AF-2 in the LBD facilitates transcriptional activation by ERα. Growth factors involved in the MAP kinase pathway controls the activity of AF-1^9^, while AF-2 function is responsive to the ligand binding^10^. Agonist’s binding stimulates AF-2 activity, whereas antagonist’s binding does not^11^. Among the ERs, the ERα subtype drew attention with constructive evidences, because it showed the change in the endocrine function due to its binding to xenoestrogens or SERMs. ERα has six structural domains. Out of the six domains, the most intact domain is the central DNA- binding domain (DBD) along with the LBD. Twelve α-helices (H1-H12) and a beta-hairpin constitute the LBD of ERα (Fig. 1a). The H12 of LBD adopts discrete ligand-dependent conformation, accountable for the receptor activation and plays the crucial molecular switch (Fig. 1b,c)^12,13^. Upon estrogen binding, the LBD transforms into an active conformation, in which H12 rests across H3 and H11 and forms an indentation to accommodate co-regulator binding. The interaction of LBD with an antiestrogenic inhibits receptor activation^14^ because H12 migrates from its natural position and causes the distortion of co-regulator binding indentation.

Phytoestrogens, are a group of naturally occurring compounds present in plants. They have the ability to bind to the ERs and to stimulate estrogen-dependent transcription^15^. Crystallographic studies show that the 4-hydroxyl group on the B ring of isoflavones mediates binding to ERs. Phytoestrogens are only weakly estrogenic; their activity is 1/100 times lower than that of E_2_^16^.

**Figure 2:**
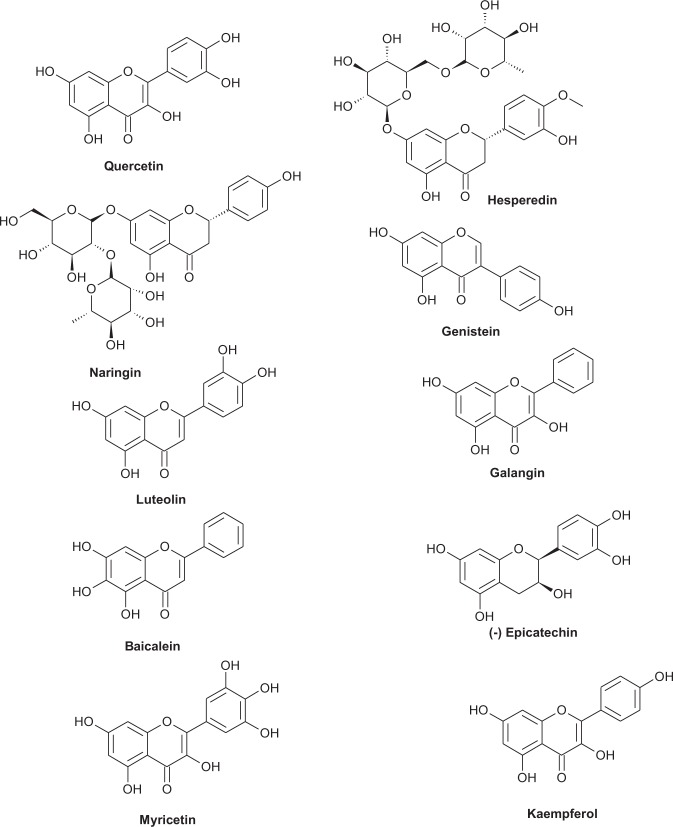
Chemical structure of flavonoids investigated for estrogenic and antiestrogenic potential with human estrogen receptor ERα.

In the ground of medicinal chemistry, the rich repertoire of flavonoids is available. They have proved meaningful potential to preclude the spread of various diseases such as cancer and diabetes^17^. These molecules are still needed to be more explored to establish as agonist or antagonist for ERs. Huang *et al*.^18^ reported SAR of flavonoids with ERγ and Suetsugi *et al*.^19^ demonstrated flavone and isoflavone as the agonist to estrogen-related receptors. Zand *et al*.^20^ reported the estrogenic, androgenic and progestational activities of flavonoids. Grande *et al*. identified homoisoflavones from *Leopoldia comosa* as ligands of ERs^21^. Recently, Jameera *et al*. focused light on the role of ERs’ agonist, the antagonist in breast cancer therapy^1^. Ng *et al*. focused on agonist and antagonist property of endocrine disrupting chemicals (EDC) by competitive molecular approach^22^. Chakraborty *et al*.^23^ published role of resveratrol as antagonist and partial agonist on ERα. We selected some most promising flavones (quercetin, myricetin, kaempferol, luteolin, baicalein, galangin), isoflavones (genistein for comparison), flavanones (naringin, and hesperidin) and flavanol (epicatechin) for our experiments (Fig. 2), although some data are already reported in literature for their agonist and antagonist potential^24–30^. The article has been reported for the first time that some of the flavonoids have binding affinity for both 3ERT (antagonist pocket) and 1GWR (agonist pocket) irrespective of their estrogenic or antiestrogenic potential using Schrodinger Maestro software. To overcome ambiguity, we performed molecular dynamics on protein-ligand systems to check for stability and protein-ligand contact maps for the complete 50 ns using Desmond software. The estrogenic/antiestrogenic activity of flavonoids was examined using a cell viability assay in ER-positive MCF-7 cells. Estrogen response element (ERE)-containing luciferase promoter construct (3xERE/3XERRE-luciferase) was used to analyse the estrogenic/antiestrogenic potential of flavonoids. Finally, the effect of flavonoids on the expression of PR (progesterone receptor), a well established estrogen regulated gene was analyzed by Western blotting.

## Results

### Molecular modelling

We selected two ERα ligand binding domains (LBD) 3ERT and 1GWR from RCSB site. 3ERT is the crystal structure of the human ERα-LBD in complex with 4-hydroxytamoxifen (OHT), which is an active metabolite of TM (well-known SERM). 1GWR is the crystal structure of human ERα-LBD in complex with E_2_, which is an endogenous agonist. Previously, we presumed that the ligand which will bind to 3ERT effectively might behave as an antagonist whereas which will interact significantly with 1GWR may function as an agonist. Surprisingly, we found that most of the flavonoids selected for the studies bind to both 3ERT and 1GWR (except naringin and hesperidin, which only bind with 3ERT). Their docking scores were also obtained closer to the standard ligands OHT and E_2_ (Table 1).

**Table 1:** Docking scores of standards and naturally occurring flavonoids with 3ERT and 1GWR along with the interacting residues of 3ERT and 1GWR.

S.No	Compound	3ERT (DS)	1GWR (DS)	3ERT (Res)	1GWR (Res)
1	TM	−10.468	−10.254	Thr347, Asp351, Glu353, Arg394, Trp383	Thr347, Glu353, Arg394,
2	E_2_	−9.872	−11.292	Ala350, Glu353, Arg394, Gly420, Met421	Glu353, Arg394, Phe404, Hie524,
3	OHT	−12.149	DNB	Asn348, Asp351, Glu353, Arg394, Leu428	DNB
4	RAL	−12.786	DNB	Thr347, Asp351, Glu351, Arg394, Trp383	DNB
5	ICI182,780	−9.876	DNB	Leu346, Thr347, Asp351, Glu353, Met388	DNB
6	GW5638	−9.170	−9.210	Arg394, Leu387, Leu 428, Gly 390 Met 421	Arg394, Leu387, Leu 428, Gly 390, Met 421
7	Gw7604	−10.486	DNB	Glu353, Arg 394	DNB
8	Quercetin	−9.347	−10.136	Glu353, Leu391, Arg394, Thr347	Glu353, Arg394, Phe404, Met421, Hie424
9	Hesperidin	−10.82	DNB	Thr347, Leu384, Met343, Val534, Leu536, Cys530	DNB
10	Naringin	−11.18	DNB	Met343, As351, Leu387, Leu521. Leu536	DNB
11	Genistein	−8.180	−9.062	Leu387, Glu353, Arg394,	Leu346, Leu387, Glu353, Arg394, Phe404, Gly521, Hie524
12	Luteolin	−7.340	−8.594	Thr347, Asp351	Leu387, Glu353, Arg394, Phe404, Ile424, Hie524
13	Galangin	−7.668	−8.091	Glu353, Arg394	Leu346, Phe404, Gly521, Hie524
14	Baicalein	−11.147	−9.907	Leu346, Glu353, Arg394, Gly521	Leu346, Glu353, Met388, Arg394, Phe404, Gly521, Hie524
15	Epicatechin	−7.548	−7.847	Glu353, Arg394	Glu 353, Leu387, Phe404, Gly521
16	Myricetin	−9.498	−9.9 77	Thr347, Asp351, Glu353, Arg394	Leu346, Leu387, Glu353, Arg394, Phe404, Hie524
17	Kaempferol	−9.867	−9.252	Asp351	Glu353, Arg394, Phe404, Hie524

**Figure 3:**
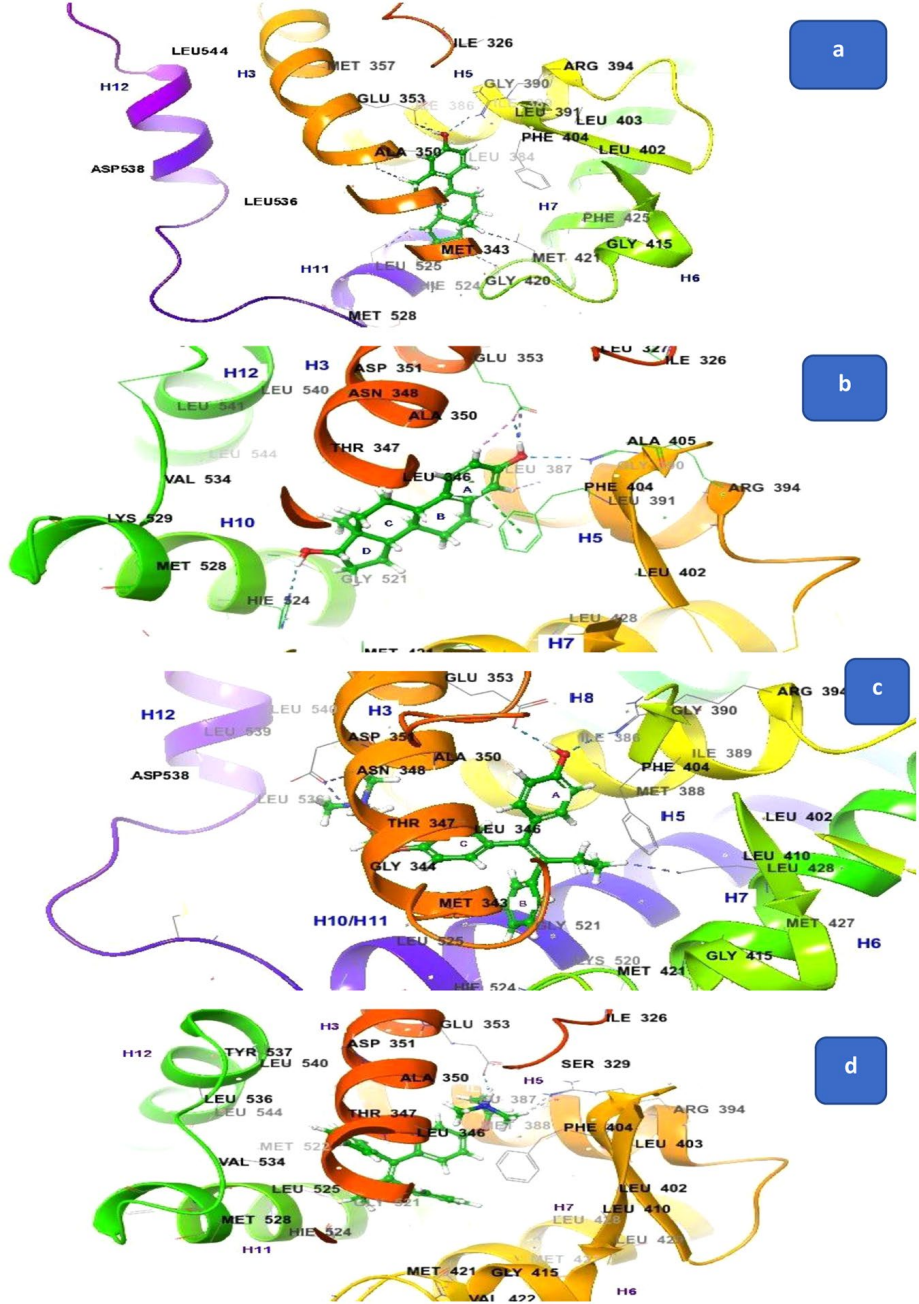
Interactions of standard TM and E_2_ with 3ERT and 1GWR (**a**) E_2_ with 3ERT; (**b**) E_2_ with 1GWR; (**c**) OHT with 3ERT; (**d**) TM with 1GWR.

To clear our perplexities, the docking studies of TM and E_2_ with both 3ERT and 1GWR were performed, and we found that both TM and E_2_ showed binding with 3ERT and 1GWR (Fig. 3), but OHT, RAL, GW7604, and ID182,780 didn’t dock with 1GWR. To understand this unusual behaviour of these ligands we followed the work published by the Greene’s group^8^. According to the evidence reported in literature^8^, it is quite clear that ligands E_2_ and OHT attach at the identical site within the core of the LBD of ERα, only the difference with that each of these ligands brings an altered conformation of H-12. The observations derived from the docking studies also revealed the same results (Fig. 3). In the E_2_ -LBD complex, H-12 packs against H-3, 5/6 and 11 in a conformation that was found similar with the other agonist diethylstilbestrol, whereas H-12 in OHT-LBD complex binds in hydrophobic groove with the residues of H-3 and 5. This significantly unlike orientation of H-12 partially buried residues in the groove that are essential for AF-2 activity, indicating that OHT acts as an antagonist and blocks the AF-2 activity by disrupting the topography of the AF-2 surface.

It was noticed from the interaction analysis, which displayed in the Fig. 3, the H-12 conformation was modified significantly, and some similarity pattern was observed in the binding of E_2_ with 3ERT and 1GWR. The hydroxyl group of ring A interacted with Glu353 and Arg394 of 3ERT and 1GWR, and the hydroxyl group of five-membered ring D (Fig. 3a,b) showed binding with Hie524. This type of similarity was not observed when antiestrogenic compounds bound with 3ERT and 1GWR. The binding image of TM-1GWR showed that the side chain of TM formed bonding with Glu353 and Arg394, whereas in the OHT-3ERT complex (Fig. 3a,b) the side chain of OHT interacted with Asp351. The hydroxyl group attached to the phenyl ring (A) formed bonding with Glu353 and Arg394.

**Figure 4:**
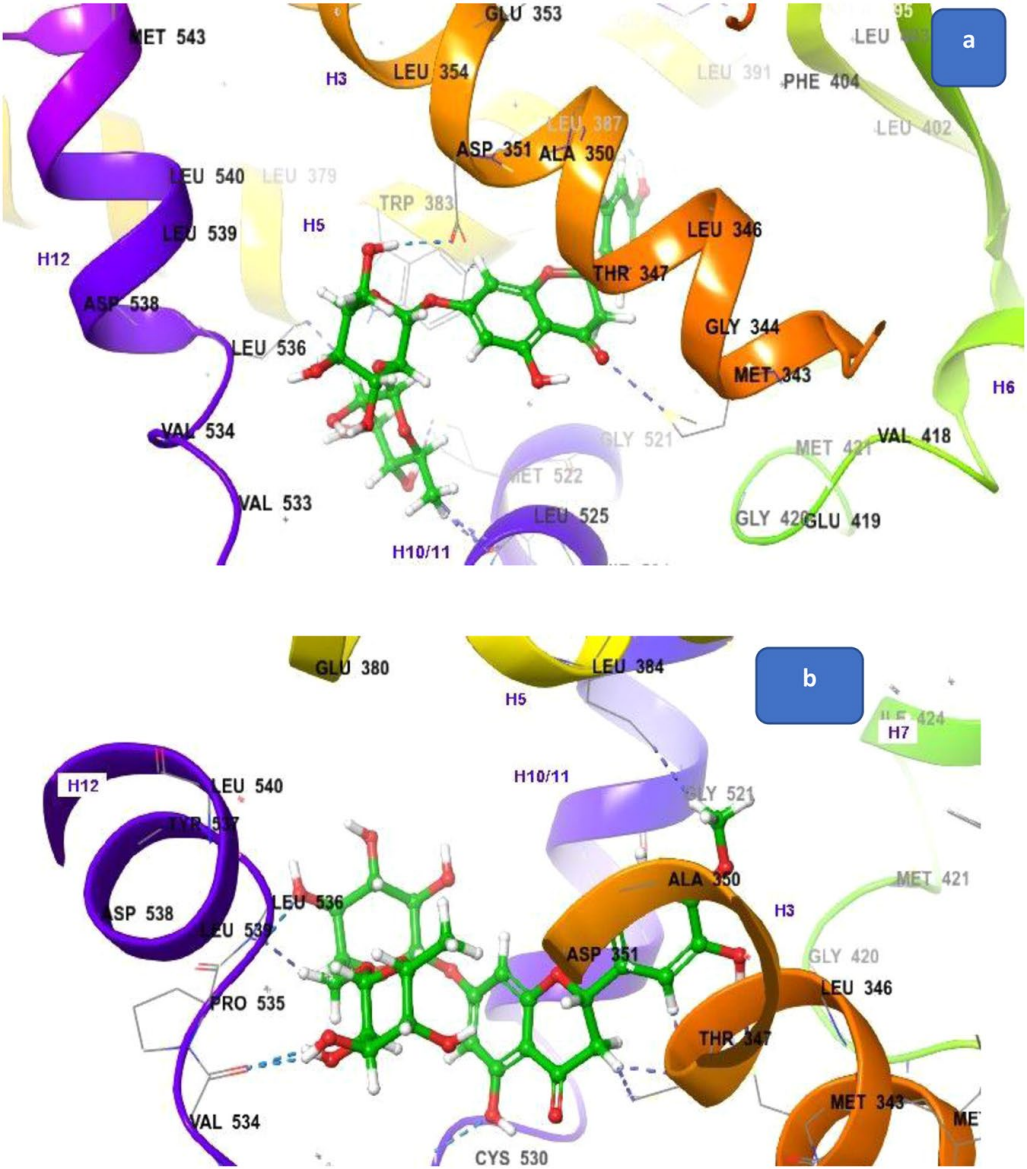
Docking of naringin and hesperidin with 3ERT (**a**) naringin with 3ERT (**b**) hesperidin with 3ERT.

Naringin showed binding with the pocket of 3ERT only in a different manner. Its sugar moiety interacted with Leu536 of H-12. The phenyl ring attached at C-2 carbon of flavanones was embedded between H-3 and 5. The hydroxyl group of this ring formed a hydrogen bond with Leu397. The CH_2_OH of pyranose ring formed a hydrogen bond with Asp351 (Fig. 4a). The carbonyl group of naringin interacted with Met343.

Hesperidin also bound only to 3ERT. The sugar moiety interacted with the residues of H-12 such as Leu536 and also with the residues Cys530 and Val534 of the turn between H-11 and 12 (Fig. 4b). Since naringin and hesperidin only interacted with 3ERT and also with H-12 residue, whose conformation is more important for antiestrogenic activities. These two flavanones can be considered as antiestrogenic.

We found rest of the flavonoids considered in this piece of work such as quercetin, myricetin, kaempferol, galangin, epicatechin, luteolin, baicalein, genistein showed docking to both 3ERT and 1GWR (supplementary file Supplementary information -I). Baicalein, epicatechin and genistein interacted with similar residues of 3ERT and 1GWR, whereas rest of the studied compounds such as, kaempferol, myricetin, galangin interacted with different residues of 3ERT and 1GWR. Their orientation also changed in active site pocket of 3ERT and 1GWR. Therefore, molecular dynamics of some compounds was performed for better understanding.

### Molecular dynamics study

Explicit Molecular Dynamics study for a period of 50 ns indicated that protein-ligand complex was stable and compatible with each other. The RMSD of the protein backbone plots (supplementary file) for 1GWR against E_2_, baicalein, epicatechin and kaempferol depicted minor deviations at a steady phase of not more than 1 Å. Similarly, the protein backbone plots for 3ERT against naringin and OHT had a stable backbone less than 1 Å deviations and naringin had slight upward trend but quite stable until full simulations. The protein-ligand contact analysis revealed that 3ERT protein had constant interaction with GLU353 for consistently for more than 50% of total simulation. The 1GWR protein had static stable interaction with Leu387, Phe404 and Glu353, which could be due to the difference between agonist and antagonist profiling of compounds.

### ADME/T Analysis

Physicochemical properties and ADME predictions such as Absorption, Distribution, Metabolism, Excretion, and Toxicity (ADME/T) properties of the molecules are important to understand their drug-like properties. An *in-silico* prediction of physically significant and pharmaceutically relevant properties of the ligands were performed using QikProp. The data are presented in Table 2.

**Table 2:** Evaluation of ADME properties of the naturally occurring flavonoids along with standard antiestrogenic and estrogenic molecules by Quikprop Maestro 11.2 molecular docking suite.

S.No	Compounds	Q P log Po/w (−2.0 to 6.5)	Q P log HERG (acceptable range: above −5.0)	QPP Caco(nm/s) <25-poor >500-great	Q P log BB (−3 to 1.2)	QPP MDCK (nm/s) <25-poor >500-great	Q P log Kp (−8.0 to −0.1)	Q P log K_hsa_ (acceptable range: −1.5 to 1.5)	Percentage of human oral absorption; (<25 % is poor and >80 % is high)
1	TM	6.566	−7.458	2274.795	0.380	1330.583	−1.328	1.362	100.00
2	E_2_	−4.010	−3.890	1202.597	−0.375	603.886	−2.713	0.458	100.00
3	OHT	5.832	−7.328	690.280	−0.254	366.636	−2.387	1.205	100.00
4	RAL	4.835	−7.688	119.471	−0.992	87.943	−4.119	0.993	92.432
5	ICI-182,780	7.491	−6.196	29.231	−1.426	2553.593	−1.929	1.483	71.123
6	GW5638	6.238	−4.867	210.093	−1.066	116.519	−1.288	1.005	92.080
7	GW7604	5.504	−4.604	62.465	−1.675	31.406	−2.374	0.767	77.769
8	Quercetin	0.360	−5.098	18.193	−2.416	6.509	−5.547	−0.345	51.602
9	Hesperidin	−1.353	−6.140	3.893	−4.372	1.230	−6.199	−1.212	0.000
10	Naringin	−1.348	−6.208	3.968	−4.233	1.255	−6.191	−1.157	0.000
11	Genistein	1.693	−5.059	161.82	−1.34	69.089	−3.567	−0.093	76.394
12	Luteolin	0.917	−4.985	14.797	−1.946	15.581	−4.895	−0.200	61.139
13	Galangin	1.756	−5.079	116.370	−1.558	48.376	−4.066	0.001	74.201
14	Baicalein	1.710	−5.094	164.045	−1.280	70.116	−3.643	−0.047	76.604
15	Epicatechin	0.477	−4.7	55.642	−1.854	21.791	−4.69	−0.413	60.97
16	Myricetin	0.247	−4.885	14.773	−2.452	5.197	−5.782	−0.351	49.322
17	Kaempferol	1.298	−5.085	114.388	−1.441	47.486	−3.994	−0.040	73.145

Naturally occurring flavonoids as ligands along with standard antiesterogens and estrogens; Predicted IC_50_ value for blockage of HERG K+ channels; (acceptable range: above-5.0); QPP Caco, predicted apparent Caco-2 cell permeability in nm/s. Caco-2 cells is a model for the gut–blood barrier; (nm/s)<25-poor >500-great; Q P log BB, predicted brain/blood partition coefficient; QPPMDCK, predicted apparent MDCK cell permeability in nm/s. MDCK cells are considered to be a good mimic for the blood–brain barrier; (nm/s) <25-poor and >500-great; Q P log KP, predicted skin permeability; Q P log K_hsa_, prediction of binding to human serum albumin; (acceptable range: −1.5 to 1.5); Percentage of human oral absorption; (<25 % is poor and >80 % is high).

Since on several occasions, the results of molecular modelling do not match within *in-vitro* and *in-vivo* experiments, other experimental methods such as luciferase assay, MTT assay and western blot experiments were performed to verify agonist and antagonist properties of flavonoids.

### Luciferase assay

**Figure 5:**
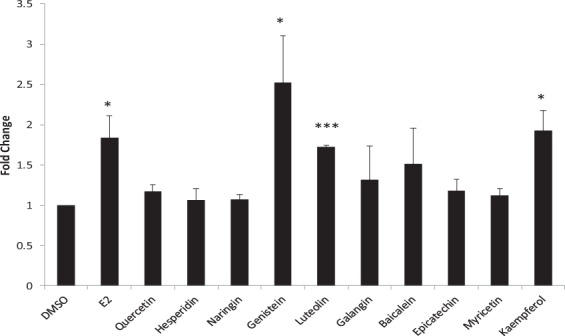
Effect of the flavonoids on ERE driven reporter gene expression. Cells were transfected with 3XERE/3XERRE- firefly luciferase and renilla luciferase constructs. Cells were treated with DMSO (0.05%), 10 nM E_2_ and 5 µM flavonoids for 24 h. Firefly luciferase activity was normalized against renilla. The normalized luciferase activity in cells treated with DMSO (vehicle control) was set to 1 and those obtained with other treatments were expressed relative to the control (Fold change). Bars represent mean Fold change ± SD (n = 3 biological replicates; each biological replicate comprising of two dishes each for control and treatment groups). Data were analysed by ANOVA followed by Tukey’s HSD (***p < 0.001, *p < 0.05).

The classical pathway of estrogen signaling involves the direct binding of ERs to specific DNA sequences called estrogen response elements (EREs)^31^. Hence, the estrogen response element (ERE)-containing luciferase promoter construct (3xERE/3XERRE-luciferase) was used to analyze the estrogenic/antiestrogenic potential of flavonoids. MCF-7 cells were transfected with luciferase reporter vector and treated with the flavonoids. Then, the luciferase activity of cells treated with compounds was measured using dual luciferase assay as described in Materials and Methods. MCF-7 cells treated with estrogen were used as positive control. As expected, luciferase activity was induced in estrogen-treated cells with respect to vehicle treated cells. Among the flavonoids, genistein, luteolin, kaempferol induced the firefly luciferase activity indicating their estrogenic potential (Fig. 5). Baicalein also induced firefly luciferase activity with a mean fold induction of 1.5. However, the induction by baicalein was not statistically significant. The raw data for luciferase assay has been provided as a supplementary file (Supplementary information-III).

### MTT assay

**Figure 6:**
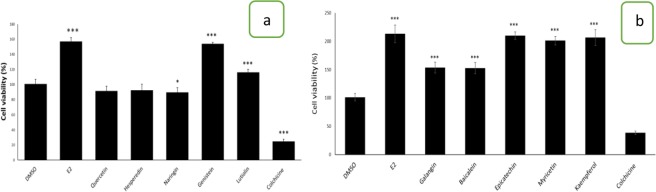
Effect of flavonoids on MCF-7 cell viability. MCF-7 cells were treated with the indicated flavonoids (5 µM), E_2_ (10 nM), Colchicine (500 nM) for a period of 120 h. Cells treated with DMSO (0.05%) served as control. MTT assay was performed as described in materials and methods. Bars represent mean percent viability ± SD. Data were analysed by ANOVA followed by Tukey’s HSD (n = 6, ***p < 0.001, *p < 0.05).

Estrogen exerts mitogenic effects on the ERα positive MCF-7 breast cancer cells^32^. Therefore, to analyze the estrogenic or antiestrogenic potential of the flavonoids, we examined their effect on MCF-7 cell viability by MTT assay. The cells were treated with the flavonoids for a period of 120 h. E_2_ treated cells was served as positive controls. Most of the flavonoids stimulated cell growth compared to vehicle treated cells. Genistein, luteolin, galangin, baicalein, epicatechin, myricetin and kaempferol increased the viability of MCF-7 cells suggesting their estrogenic potential (Fig. 6). On other hand, quercertin, naringin and hesperidin didn’t increase the viability of cells which is in coherence with our docking study.

### Western blotting

**Figure 7:**
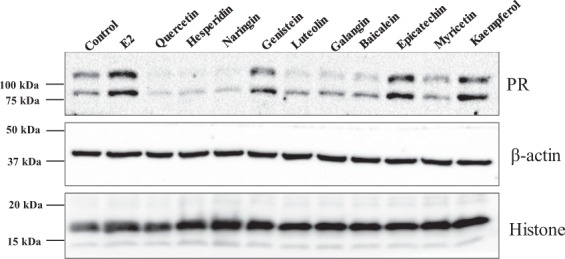
Effect of flavonoids on PR expression in MCF-7 cells. MCF-7 cells were treated with the indicated flavonoids (5 µM), E_2_ (10 nM), for a period of 24 h. Total protein was resolved in 10% denaturing SDS-PAGE, transferred to nitrocellulose membrane and probed with antibodies against PR and β-actin, histone H3. Ponceau S stained blot, white light image and chemi-luminescence images are presented in Supplementary File (Supplementary information-IV).

PR is one of the well-known estrogen-induced gene in MCF-7 cells^33,34^. Therefore, the effect of flavonoids on the expression of PR was analyzed by Western blotting. As expected, PR expression was induced in E_2_ treated cells with respect to vehicle treated control (Fig. 7). Genistein, epicatechin, kaempferol induced the expression of PR at protein level indicating their estrogenic potential (Fig. 7). Myricetin and baicalein did not induce PR expression. Reduction in PR level was observed upon stimulation with quercetin, hesperidin, naringin suggesting towards their antiestrogenic potential.

## Discussion

Phytoestrogenic flavonoids possess estrogen like activities due to some structural similarity with E_2_^20,35^. Like estrogen, they can interact with ERs at lower affinity and stimulate gene expression. ERs distinguish the structural difference of the ligand and execute the activation function^36–38^. Most of the work previously published on hormone-like property of flavonoids include, using recombinant yeast strain^30^, chemopreventive potential of baicalein on E_2_ induced transformation of breast epithelial cells^24^, biphasic effect of kaempferol on estrogenicity in human breast cancer cells^25^. In our studies we found that most of the flavonoids interacted with both 3ERT and 1GWR, only their orientation and interactions changed with different amino acid residues. The flavanones with sugar moieties interacted with H-12 of ERα. Antiestrogens could not bind to 1GWR during molecular docking.

To investigate the stability of the ligand-protein system in aqueous solution, the docking conformation (baicalein, epicatechin, kaempferol and E_2_ with 1GWR, naringin and OHT with 3ERT) generated by Maestro were taken as initial conformation for MD simulations. Explicit MD study for a period of 50 ns indicated that protein ligand complexes were stable and compatible with each other. The RMSD of the protein backbone plots for 1GWR against E_2_, epicatechin, kaempferol and baicalein showed minor deviations at a steady phase. The 3ERT protein against OHT and naringin had a stable backbone. Naringin had slight upward trend but quite stable until full simulations. The protein-ligand contact analysis revealed that 3ERT had constant interaction with GLU353 for consistently for more than 50% of total simulation; 1GWR protein had static stable interaction with Leu387, Phe404 and Glu353 which could be the difference between agonist and antagonist profiling of compounds.

Challenges are escalating in the pharmaceutical research, which need to be addressed with respect to toxicological effects such as circumventing interactions with hERG (human Ether-a-go-go-Related Gene) as well as problems associated with human oral absorption and gastrointestinal absorptions. Computational analyses to predict the pharmacokinetics of the molecules provide meaningful information regarding absorption, distribution, metabolism, elimination/excretion and toxicity. ADMET defines the druglike properties of molecules. In Table 2, ADME properties of tested compounds were compared with known agonist and antagonist compounds. In flavonoid class of compounds genistein is well known for its agonist properties with human ERα. Baicalein, kaempferol and epicatechin showed all values such as QP log Po/w, Q P log HERG, QPP Caco, QP log BB, QPP MDCK, Q P Log Kp, Q P log K_hsa_ and percentage of human oral absorption in the acceptable range and close to genistein. Naringin and hesperidin didn’t show good human oral absorption.

In order to validate the docking results, *in vitro* experiments such as dual-luciferase assay, MTT assay, and western blotting were performed. ERs are the key component in the estrogen signaling classical pathway. Estrogen signaling involves the direct binding of ERs to a specific sequence in the DNA called estrogen response elements (EREs). Hence, ERE -containing luciferase promoter construct (3xERE/3XERRE-luciferase) was used to analyse the estrogenic/antiestrogenic potential of flavonoids. ER upon stimulation by E_2_ binds to 3XERE in luciferase construct which in turn increases the expression of luciferase. Among the flavonoids, hesperidin and naringin didn’t show increased expression of luciferase while kaempferol, luteolin, baicalein exhibited estrogenic potential in the same way as genistein but weaker than it (Fig. 5).

When MTT assay was used as an indicator of estrogenic/antiestrogenic effects, except quercetin, hesperidin, and naringin, all the flavonoids increased the viability of MCF-7 cells. The inability of quercetin, hesperidin, and naringin to increase cell viability correlates with the failure of these flavonoids to induce PR expression. However, luteolin, galangin, baicalein, and myricetin, did not induce the expression of PR, while they had a positive effect on cell viability. Therefore, kaempferol and epicatechin could be considered as estrogenic at 5 µM concentration, and various concentration of luteolin, baicalein, and myricetin should be tested to corroborate their estrogenic/antiestrogenic potential.

## Conclusion

Molecular docking is a virtual screening which helps us to understand the interactions of the ligand with the receptors and to find out the effectiveness of binding. To support the data with experiments also provide the invincibility to the results. Phytoestrogens are effective and mostly safe because their daily consumptions in our diet improve our strength to fight with the disease.

Though baicalein had shown promising binding with 3ERT and 1GWR and its docking score, MD studies, and luciferase assay results supported for estrogenic potential, but it couldn’t show promising cell viability in MTT assay. In western blot, the expression of PR was weak in comparison to genistein. Therefore, it may have dual behaviour of estrogenic and antiestrogenic which could be confirmed using different concentration. Epicatechin, myricetin, luteolin, and kaempferol had different ways of interactions with 3ERT and 1GWR, but they have shown effective estrogenic potential at 5 µM concentration in luciferase, MTT and Western blot assays. We couldn’t deny the effectiveness of virtual screening, but wet lab experiments provide more assured results. Naringin and hesperidin showed only interactions with 3ERT and didn’t show increase in luciferase expression. Their antiestrogenic potential should be further tested and confirmed. Therefore, the work carried out by us may provide a better understanding of docking and the experimental results of the estrogenic and antiestrogenic potential of flavonoids. The richness of naturally occurring flavonoids should be more explored at different concentrations to identify the best lead compounds to establish them as SERMs.

## Methods

All the naturally occurring flavonoids were procured from Sigma Aldrich. Their purity was checked with HPLC (Waters) using a C_18_ column. The details are mentioned in the supplementary file (Supplementary information-V).

The 3D structures of ERα complexes with an agonist and an antagonist, i.e. E_2_ (PDB ID: 1GWR) and OHT (PDB ID: 3ERT), respectively, were chosen as the ideal docking target proteins. The ideal proteins were selected to define the agonist, antagonist and SERM behavior of selected naturally occurring flavonoids because 3ERT has ligand binding domain to show antagonist behavior of TM and 1GWR has ligand binding domain of agonist or estrogenic potency of E_2_.

### Protein preparation

The selected PDB files of 3D ERα structures for docking studies of naturally occurring flavonoids as agonists (1GWR) and antagonists (3ERT) were downloaded from RCSB site (www.rcsb.org) and pretreated prior to the docking calculation with the help of the Protein Preparation Wizard icon of Maestro 11.2 program by Schrodinger. The guidelines to use the protein preparation wizard was taken by Maestro online tutorial.

### Ligand preparation

The twenty naturally occurring molecules along with known antiestrogenic such as TM, RAL, GW5638, GW7604, and ICI182,780 were selected based on their bioactive data^39^. The E_2_ was considered as the standard agonist to estrogen receptor 1GWR. The ligands were prepared as per the guidelines of Maestro ligand preparation wizard. Energy minimization and optimization were performed using ‘optimized potential for liquid simulations’ (OPLS) force field. Epik was used for generating the tautomeric and protonation state at Biological pH^40^.

### Estrogen receptor grid generation

GLIDE molecular docking supports one ligand to interact with the X-ray crystal structure of the target protein for evaluation of the active site receptor grid. Receptor grid dependent molecular docking helps the ligands to bind in many possible conformations. Docking grids for both protein structures 1GWR and 3ERT were created using the receptor grid generation option of Maestro.

### GLIDE molecular docking

After concocting the ligand and protein and specifying the grid on the active location of the protein, molecular docking measures were achieved. The docking scores were obtained. GLIDE module of the XP visualizer analyzed the specific ligand-protein interactions. The naturally occurring flavonoids and standard ligands were docked with the 3D structure of ERα (PDB; 3ERT and 1GWR) with the help of GLIDE. The finest fit compounds were defined for each target by thermodynamic optimal energy value, types of interactions, the potential of bonding, and conformations^41,42^.

### Molecular dynamics study

Explicit Molecular Dynamics study was done on protein-ligand systems to check for stability and protein-ligand contact maps for the complete 50 ns using Desmond software^43^. The prepared protein-ligand complex was solvated with TIP4P solvent model using predefined Orthorhombic Box Shape and appropriate ions to neutralize the system using force field OPLS 2005. The solvated ligand-protein complex were then subjected to equilibrium runs as per default parameters of Desmond MD followed by production explicit molecular dynamics study for a period of 50 ns using Nose Hoover Chain thermostat and Martyna Tobias Klein Barostat to maintain the default temperature and pressure with NPT Ensemble; The complete results would be saved as trajectory that could be analysed for stability using RMSD (root mean square deviations) and protein-ligand contacts throughout the simulation time.

### ADME properties studies

QuikProp tool of Schrodinger 2017 was used to calculate the ADME properties of ligands which provided information about Absorption, Distribution, Metabolism, Excretion, and Toxicity (ADME/T) properties of the ligands molecules^44^. It delivered information such as QP log Po/w, QP log BB, overall CNS activity, Caco-2, MDCK cell permeability, logK_hsa_ for human serum albumin binding, percentage of human oral absorption, etc.

### Plasticware and reagents

MCF-7 cells were a kind gift from Dr. Dipak Datta, Central Drug Research Institute, Lucknow, India. Cell culture plasticware were purchased from Eppendorf AG (Hamburg, Germany). Fetal bovine serum (FBS) were from Invitrogen Corporation (USA). Cell culture media and charcoal-stripped FBS (csFBS), trypsin, penicillin and streptomycin, nitrocellulose membrane, and MTT were purchased from HiMedia (Mumbai, India). Lipofectamine 3000 was from Invitrogen Corporation (USA). Progesterone receptor (PR) antibody (Catalog No.-3176S) and anti-rabbit secondary antibody (Catalog No.-7074S) were purchased from Cell Signaling (Beverly, MA). β-Actin antibody (Catalog No.- AM4302) was purchased from Invitrogen Corporation (USA). Histone antibody (Catalog No.- BB-AB0055) was from BioBharati Life sciences (India). 3xERRE/ERE-luciferase was a kind gift from Rebecca Riggins (Addgene plasmid # 37852). pRL-SV40P was a gift from Ron Prywes (Addgene plasmid # 27163). 17β-estradiol (E2) and colchicine were from Sigma Aldrich (USA). Clarity Western ECL Substrate was purchased from Bio-Rad (USA). Dual luciferase assay kit was procured from Promega Corp (USA). Routine laboratory buffers, solvents, and salts were either from Merck (Mumbai, India) or SRL (Mumbai, India).

### Cell culture

MCF-7 cells were routinely cultured and expanded in phenol red-containing DMEM supplemented with 10% FBS, 100 units/ml penicillin and 100 μg/ml streptomycin (M1 medium). However, for treatment of cells with E2 or other ligands, phenol red-free DMEM-F12, which was supplemented with 10% csFBS, 100 units/ml penicillin and 100 μg/ml streptomycin was used (M2 medium).

### Luciferase assay

MCF-7 cells were seeded in 24-well plates at a density of 90,000 cells per well in M1 medium. After 24 hours, cells were transfected with 250 ng of 3xERRE/ERE-luciferase and 2.5 ng of pRL-SV40P using Lipofectamine 3000 for 7 hours. Media was replenished after 7 hours and cells were allowed to recover for 24 hours. Then, M1 medium was replaced with M2 medium and incubated for 4 h. Following M2 medium wash, cells were treated with 5 µM of test compounds, 10 nM E_2_ or DMSO (0.05%) for 12 hours. The cells were lysed with 100 µL passive lysis buffer. Luciferase activity in the lysate was measured using Dual-Luciferase^®^ Reporter Assay kit as per manufacturers’ instructions in GloMax^®^ 20/20 Luminometer. The ratio of activities of firefly and renilla luciferase was considered as relative luminescence unit (RLU). Fold change indicates relative RLU of compound-treated cells with respect to vehicle (DMSO) treated cells.

### MTT assay

MCF-7 cells were seeded in 96-well plate at a density of 5000 cells per well in M1 medium. After 48 hours, cells were treated with 5 µM of test compounds, E_2_ (10 nM), colchicine (500 nM) or DMSO (0.05%) in M2 medium for 120 h. E_2_-treated cells were considered as a positive control while colchicine-treated cells were considered as a negative control. Treatment media was replenished every 48 hours. The experiment was terminated by adding 100 uL of MTT reagent (5 µg/mL) per well and incubated in the dark for 3 hours. Then, MTT was removed, and formazan crystals were dissolved in 100 µL of DMSO, and the absorbance was measured at wavelength 570 nm with reference wavelength 690 nm in ELISA reader (Tecan).

### Western blotting

MCF-7 cells were seeded in 6-well plates at a density of 2 × 10^5^ cells per well in M1 medium. When the cells were 70–80% confluent, M1 medium was replaced with M2 medium and incubated for 4 h. Cells were then treated with 5 µM of test compounds, 10 nM E2 or DMSO (0.05%) for 24 hours. The cells were lysed with Laemmli buffer. Proteins were resolved in 12% SDS-PAGE and transferred to nitrocellulose membrane by the semi-dry transfer method. After blocking, the blots were probed with PR antibody, β-actin antibody or histone antibody for 2 hours. Then blots were washed and incubated with HRP-tagged anti-rabbit secondary antibody for one hour. After washing, blots were developed using Clarity Western ECL Substrate.

## Supplementary information

Supplementary File
