# Supplementary material for: Determination and analysis of agonist and antagonist potential of naturally occurring flavonoids for estrogen receptor (ERα) by various parameters and molecular modelling approach
Source: Sci Rep. 2019 May 15;9:7450. doi: 10.1038/s41598-019-43768-5 (PMC6520524; doi:10.1038/s41598-019-43768-5)
Supplement: Supplementary file 1 — Supplementary File [file 41598_2019_43768_MOESM1_ESM.pdf]

Determination and analysis of agonist and antagonist potential of naturally occurring flavonoids for estrogen receptors (ERα) by various parameters and molecular modelling approach.

Ninad V. Puranik[1,3] and Pratibha Srivastava[1,3]*, Gaurav Bhatt[2], Dixcy Jaba Sheeba John Mary[2], Anil M. Limaye[2], Jayanthi Sivaraman[4].

[1]Bioprospecting Group,

Agharkar Research Institute, G. G. Agarkar Road, Pune-411004, Maharashtra, India

[2]Department of Biosciences and Bioengineering, Indian Institute of Technology, Guwahati-781039, Assam, India

[3]Savitribai Phule Pune University, Ganeshkhind, Pune-411007

[4]Computational Drug Design Lab, School of Bio Sciences and Technology, Vellore Institute of Technology, Vellore-632014, Tamil Nadu, India

Ninad V. Puranik ninadv_puranik@yahoo.co.in He has done molecular modelling studies.

Pratibha Srivastava (Corresponding author) psrivastava@aripune.org. Idea generation, interpretation of modeling results and manuscript writing.

Gaurav Bhatt gauravbhatt.iitg@gmail.com He has done quantitative western blotting assay and MTT assay.

Dixcy Jaba Sheeba John Mary d.sheeba@iitg.ac.in She has done Luciferase assay.

Anil M. Limaye amul@iitg.ac.in; limaye.anil@gmail.com; Guided both Gaurav and Sheeba to perform the assay and interpretation of results.

Jayanthi Sivaraman jayanthi.s@vit.ac.in. She has helped in Molecular Dynamics studies.

Supplementary Information- I

Flavones which interact with both 3ERT and 1GWR

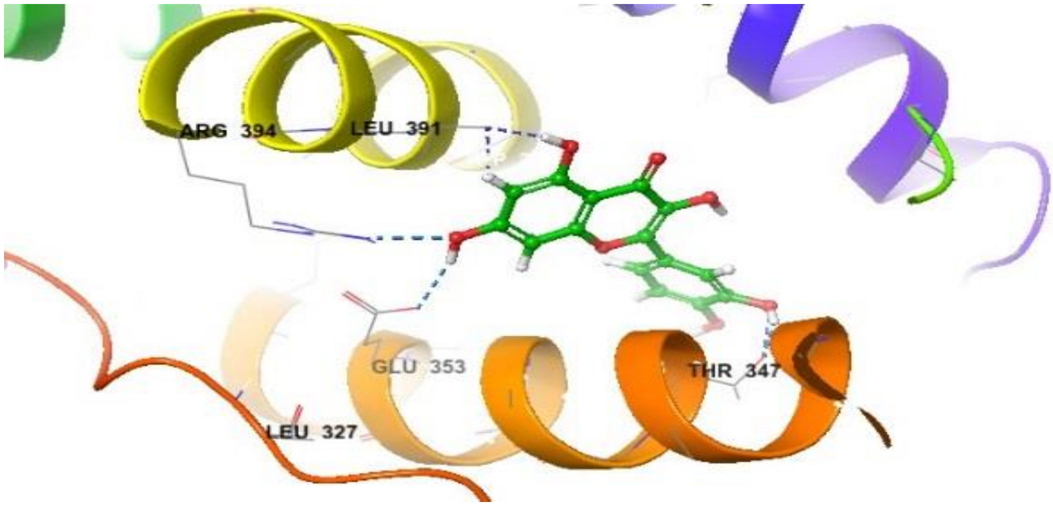

Quercetin with 3ERT

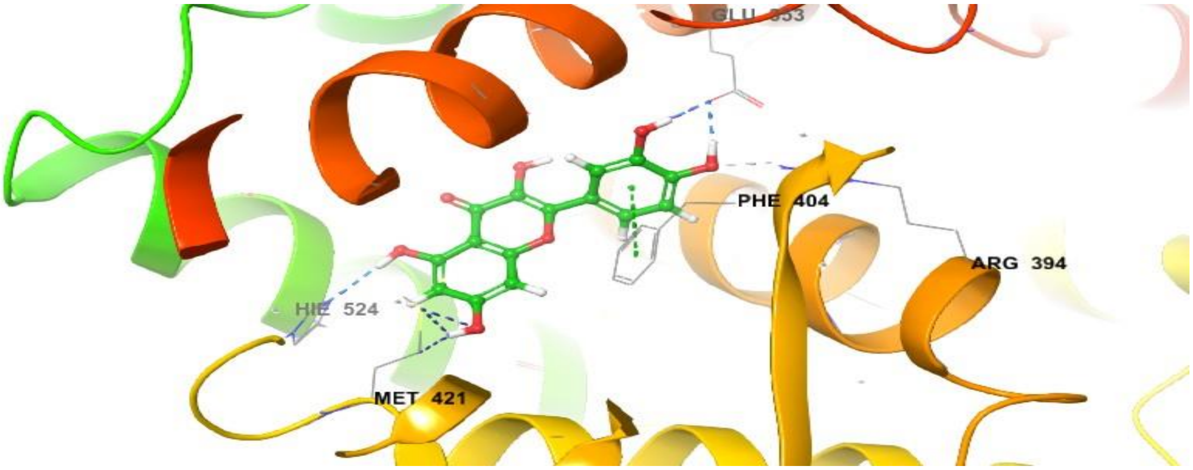

Quercetin with 1GWR

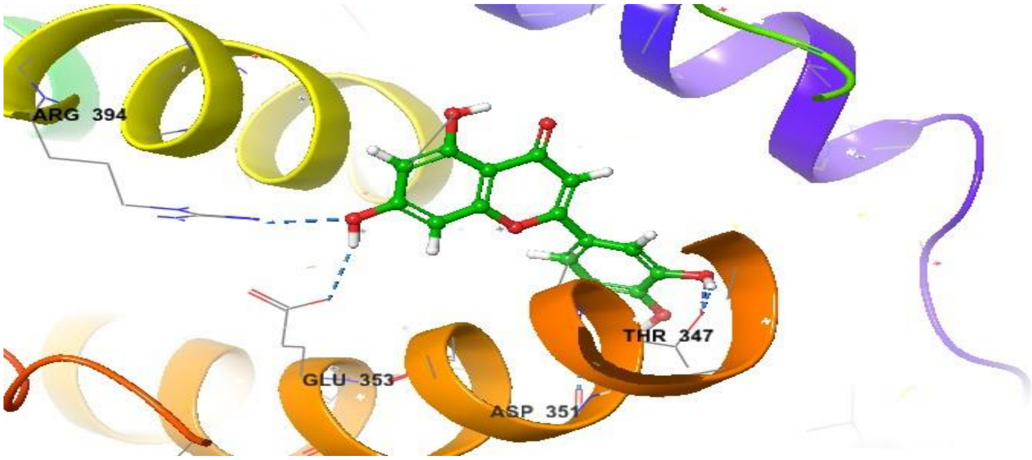

Myricetin with 3ERT

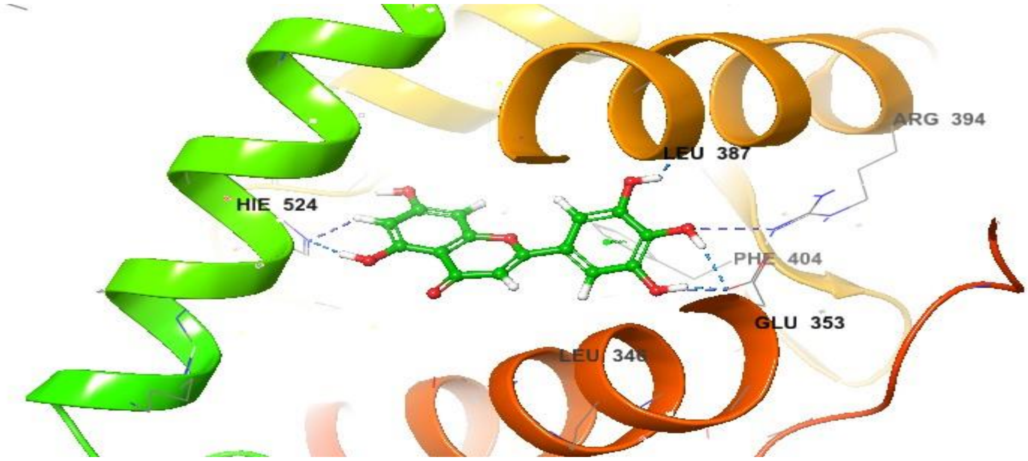

Myricetin with 1GWR

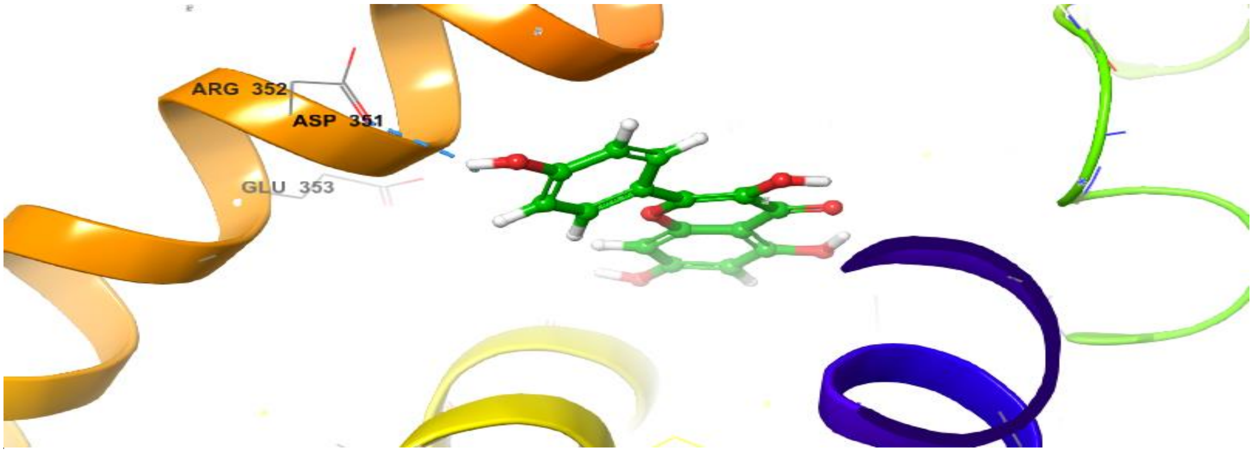

Kaempferol with 3ERT

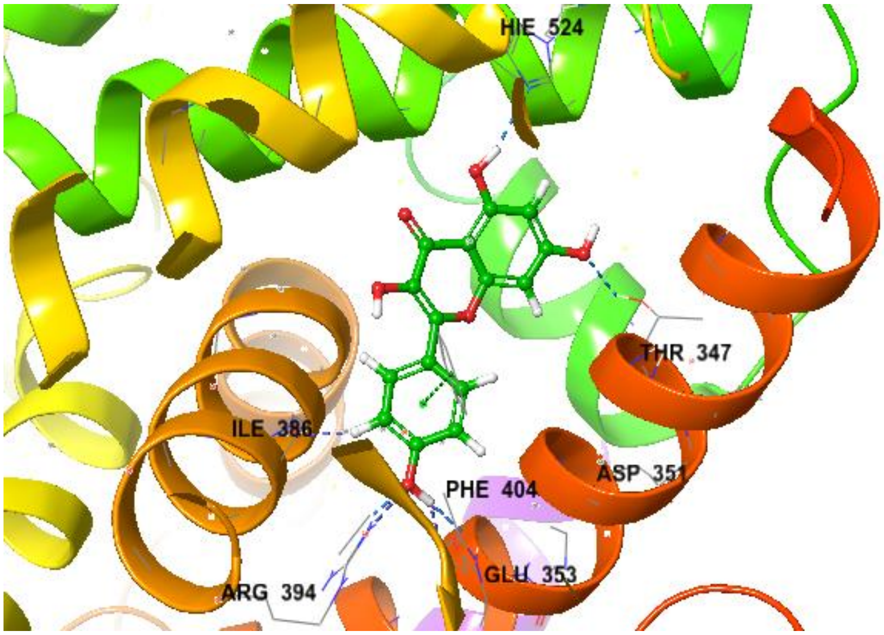

Kaempferol with 1GWR

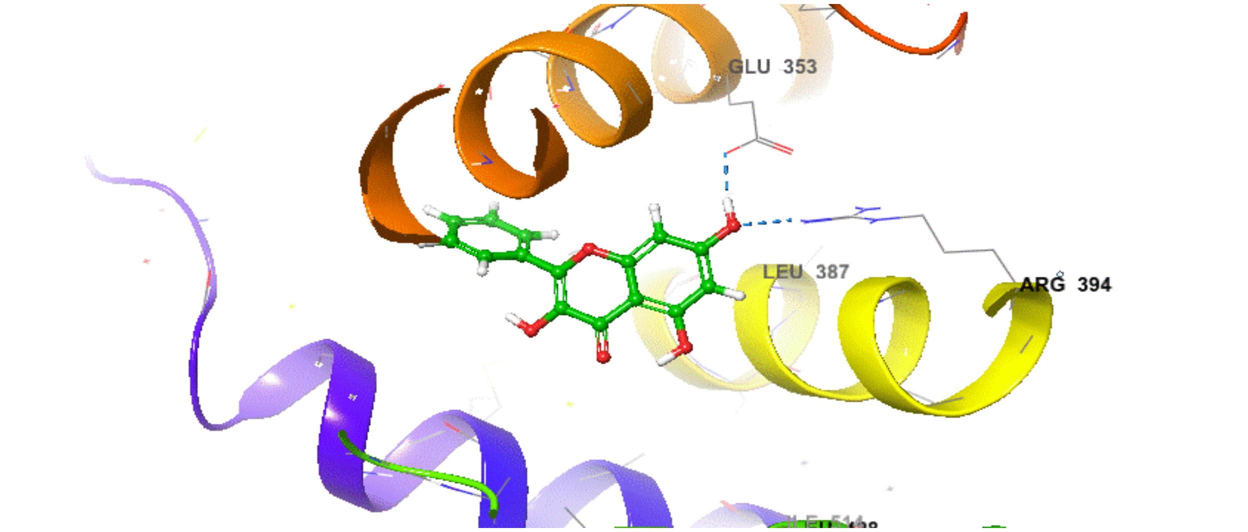

Galangin with 3ERT

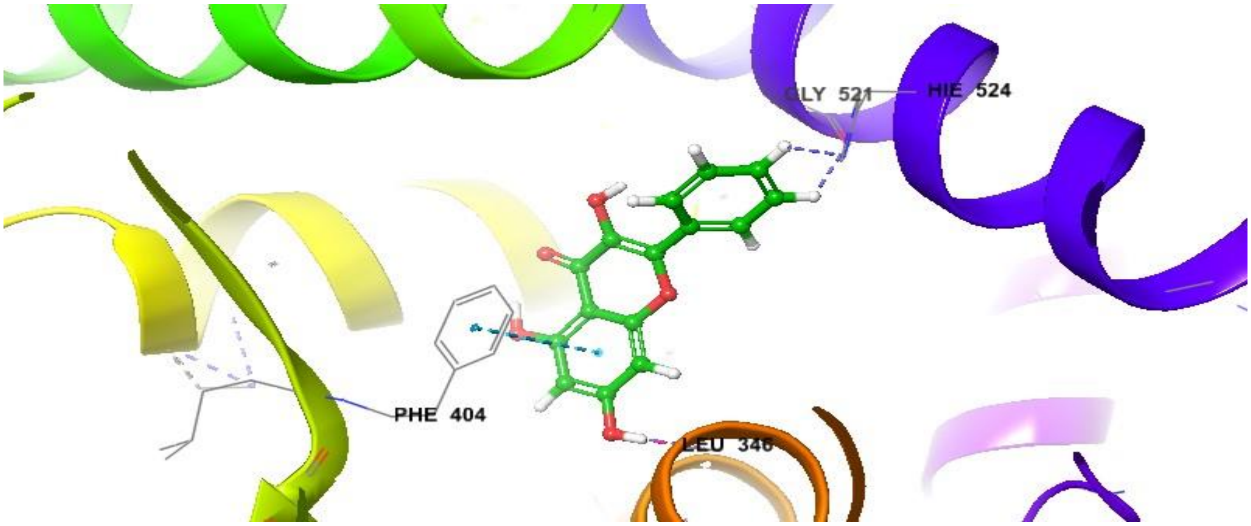

Galangin with 1GWR

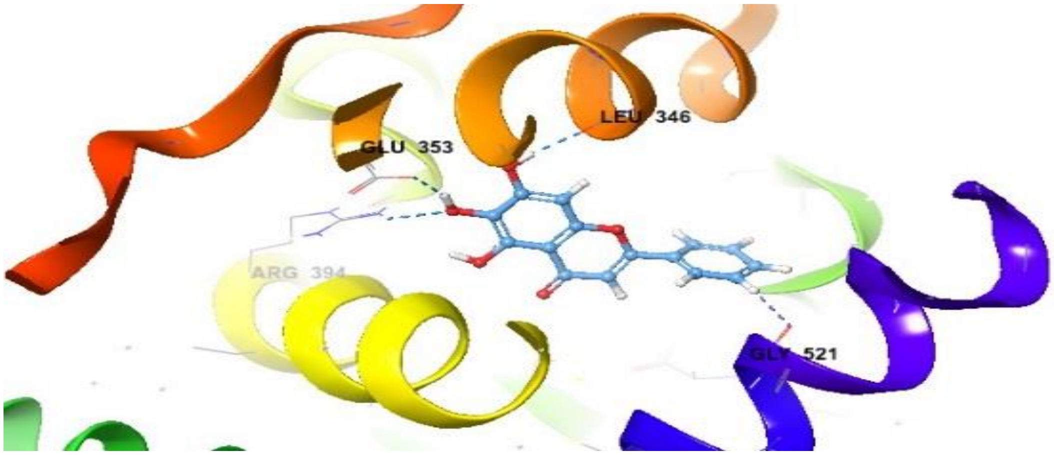

Baicalein with 3ERT

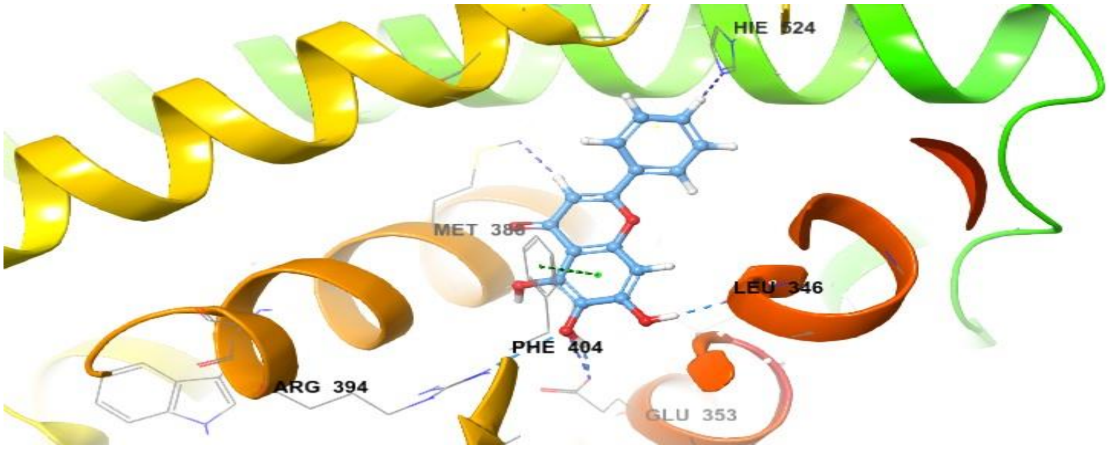

Baicalein wit 1GWR

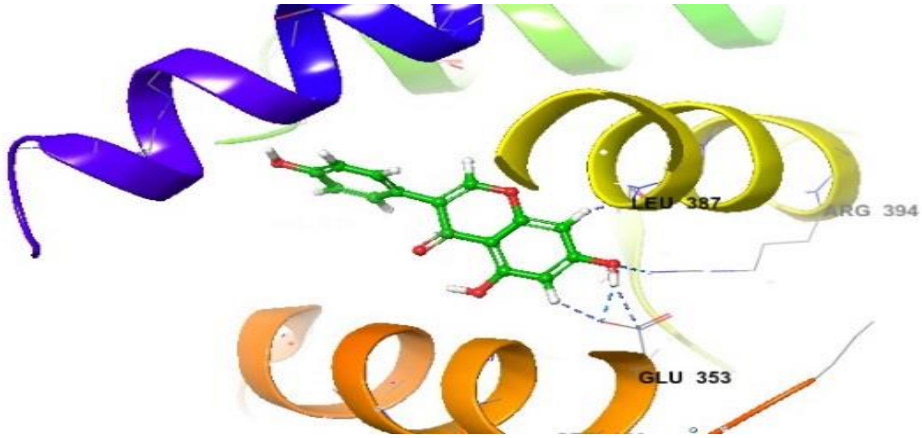

Genistein with 3ERT

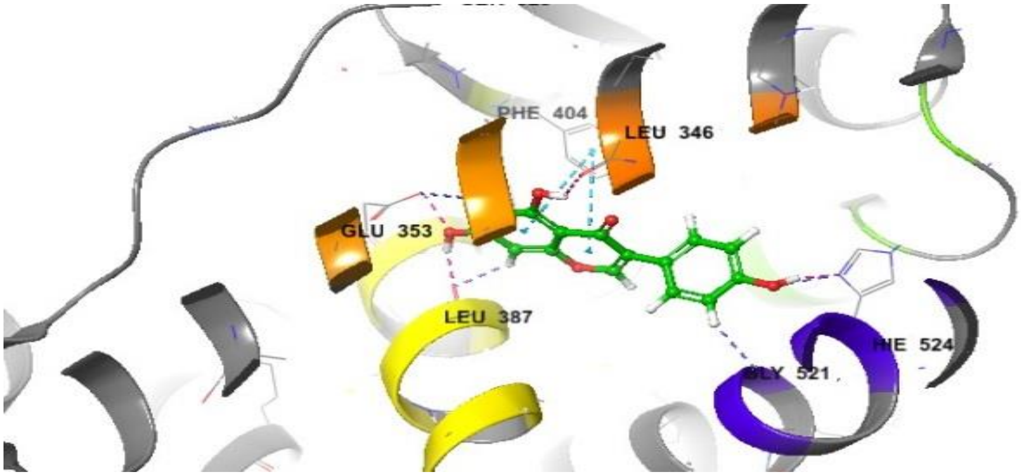

Genistein with 1GWR

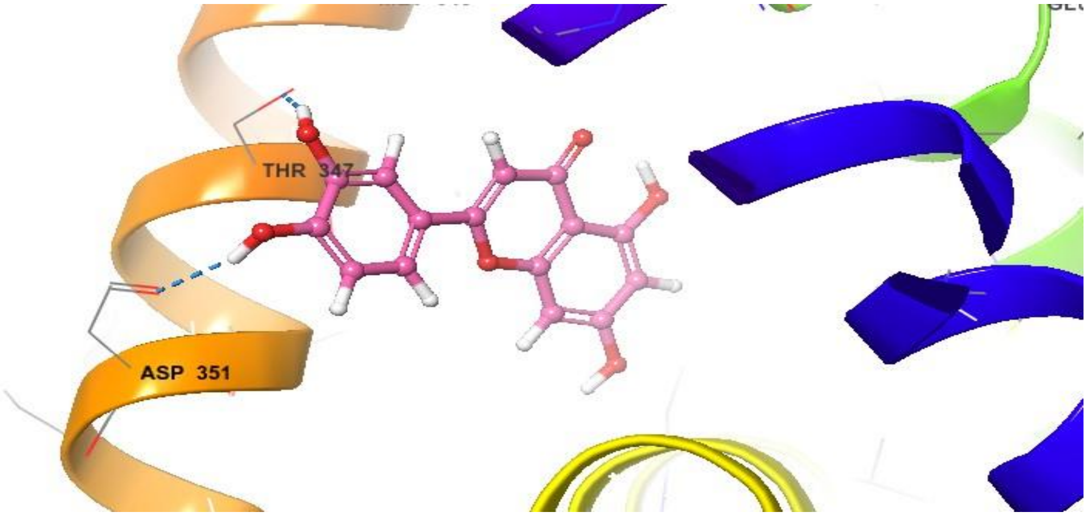

Luteolin with 3ERT

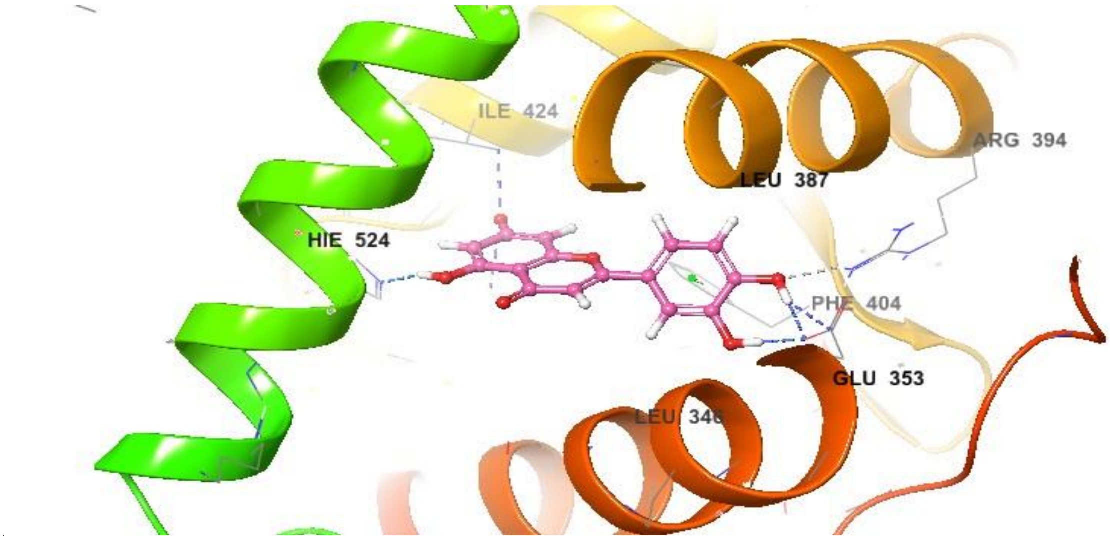

Luteolin with 1GWR

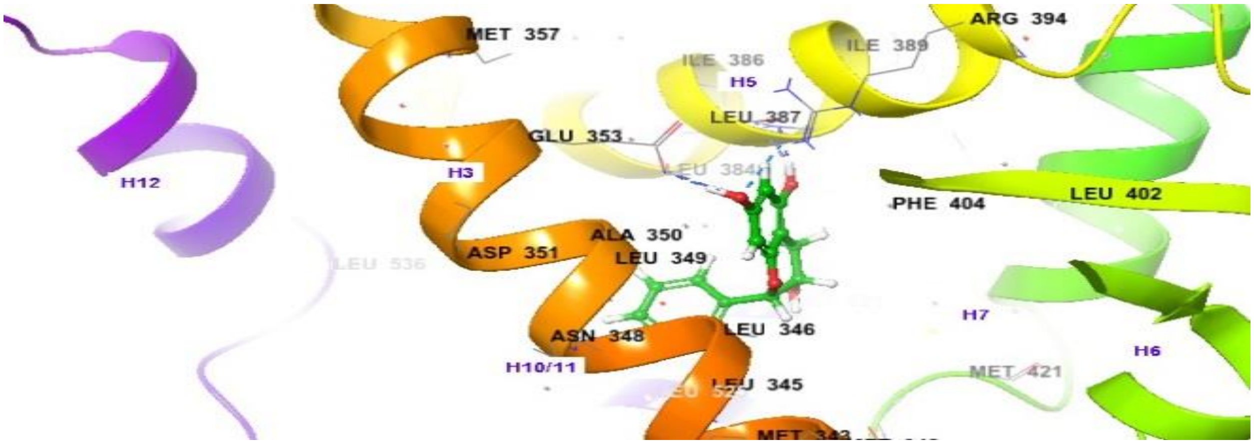

Epicatechin with 3ERT

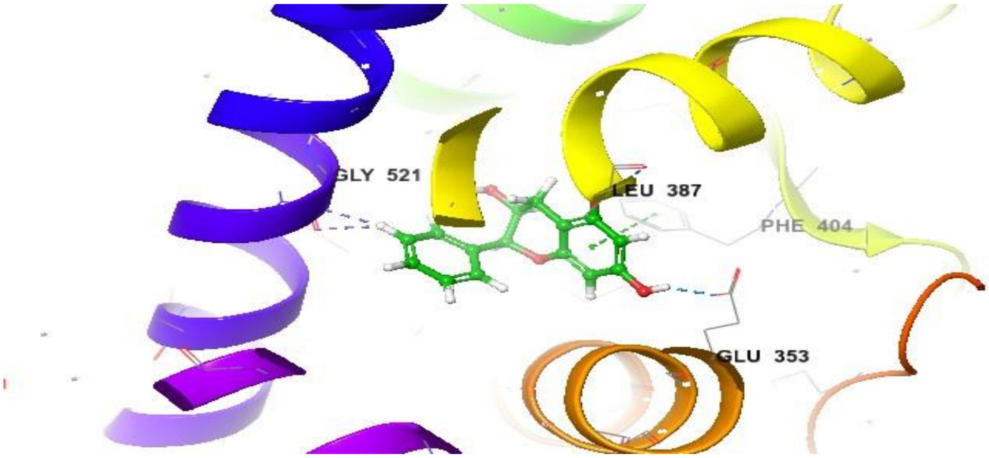

Epicatechin with 1GWR

Supplementary Information-II

Molecular Dynamics Results

Molecular Dynamics Protein Ligand Contact Map on 1GWR

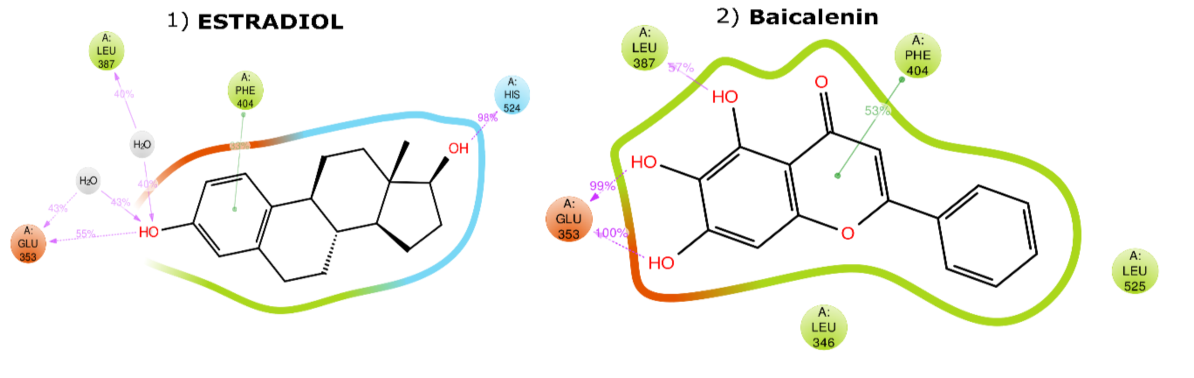

Molecular Dynamics Protein Ligand Contact Map on 3ERT

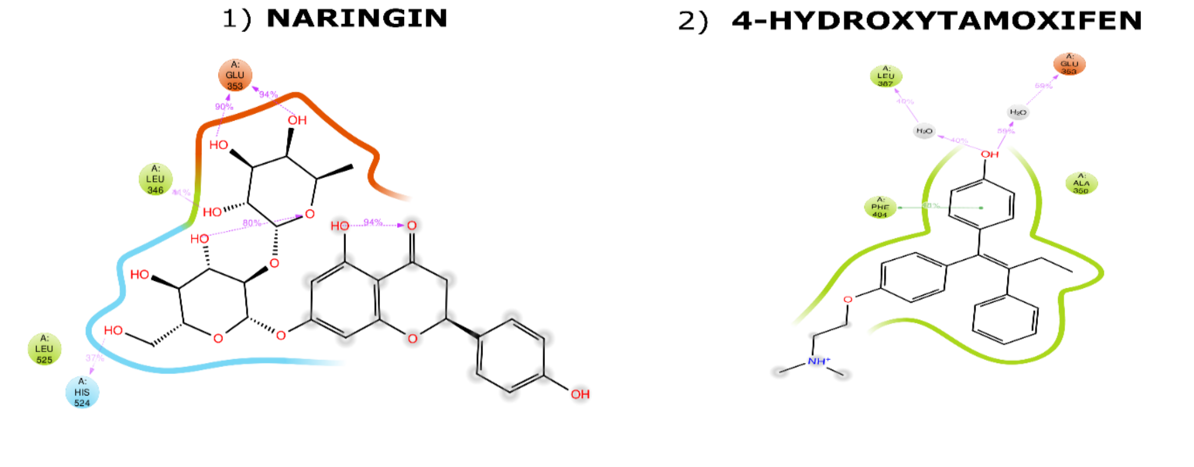

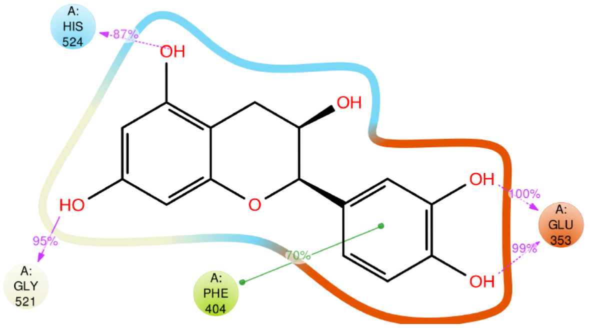

Epicatechin with 1GWR

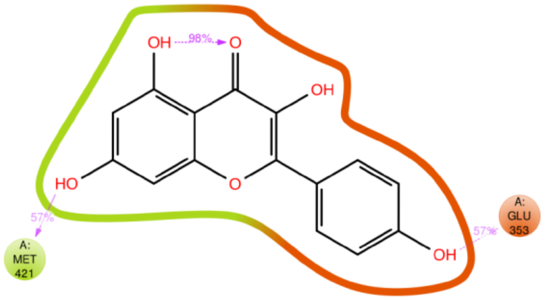

Kaempferol with 1GWR

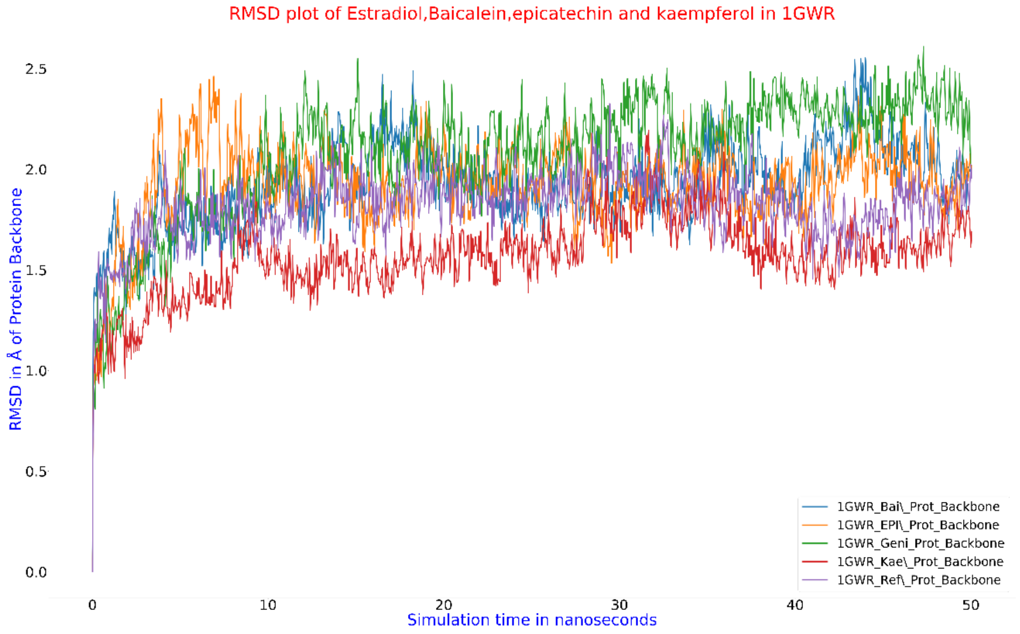
RMSD plot of Estradiol,Baicalein,epicatechin and kaempferol in 1GWR
RMSD in Å of Protein Backbone
Simulation time in nanoseconds
1GWR_Bai_Prot_Backbone
1GWR_EPI_Prot_Backbone
1GWR_Geni_Prot_Backbone
1GWR_Kae_Prot_Backbone
1GWR_Ref_Prot_Backbone
0.0
0.5
1.0
1.5
2.0
2.5
0
10
20
30
40
50

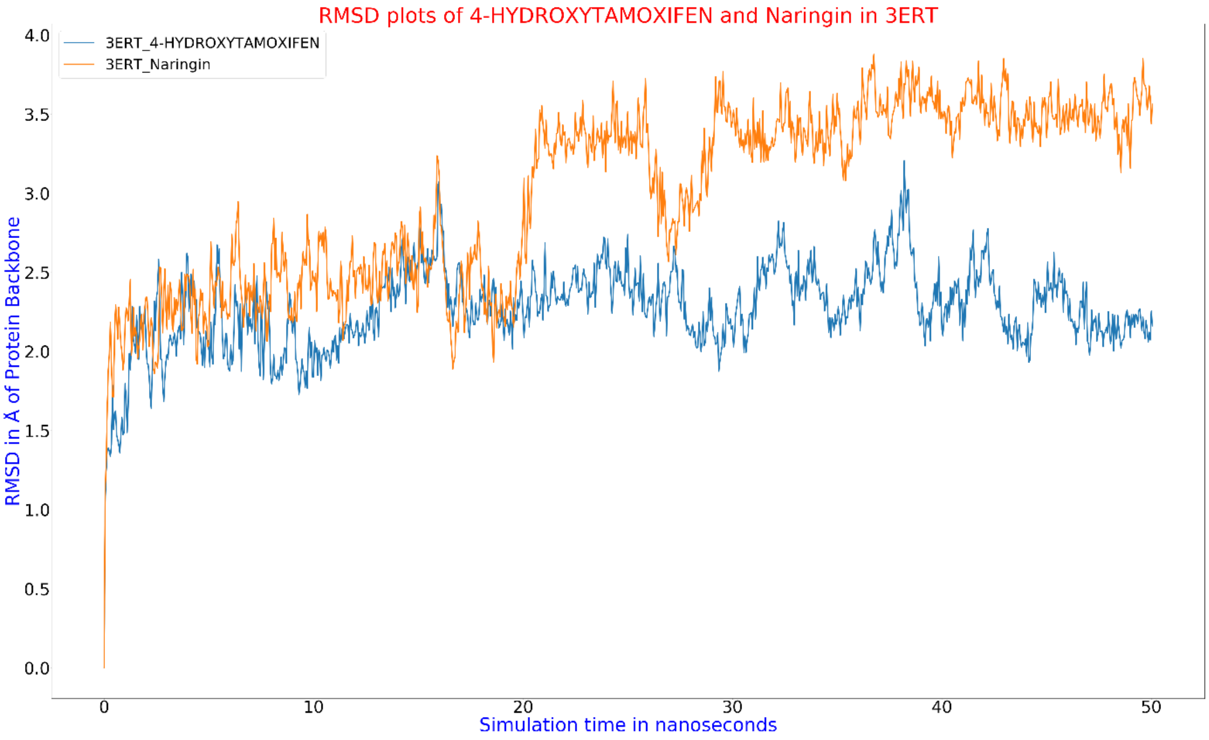
RMSD plots of 4-HYDROXYTAMOXIFEN and Naringin in 3ERT
3ERT_4-HYDROXYTAMOXIFEN
3ERT_Naringin
RMSD in Å of Protein Backbone
Simulation time in nanoseconds
0.0
0.5
1.0
1.5
2.0
2.5
3.0
3.5
4.0
0
10
20
30
40
50

Supplementary Information -III

Raw Data of Luciferase assay

	FC-BR1	FC-BR2	FC-BR3	Average	SD	p-value
DMSO	1	1	1	1	0	
E2	1.524538	1.959722	2.025365	1.836542	0.272189	0.033525
Quercetin	1.245783	1.186381	1.089003	1.173723	0.079153	0.062755
Hesperidin	1.220846	0.949293	1.025922	1.065354	0.140005	0.50368
Naringin	1.058889	1.143292	1.011338	1.071173	0.066829	0.206393
Genistein	1.881791	3.016582	2.671976	2.52345	0.581793	0.045334
Luteolin	1.730867	1.69718	1.734446	1.720831	0.02056	0.000271
Galangin	1.803039	1.101608	1.047011	1.317219	0.421617	0.322353
Baicalein	1.984276	1.102896	1.440352	1.509175	0.444702	0.185814
Epicatechin	1.124523	1.337039	1.077563	1.179708	0.138261	0.153215
Myricetin	1.148688	1.192136	1.038659	1.126494	0.079109	0.109392
Kaempferol	1.642337	2.067958	2.078536	1.929611	0.248842	0.023062

Supplementary Information -IV

Western blot gel images

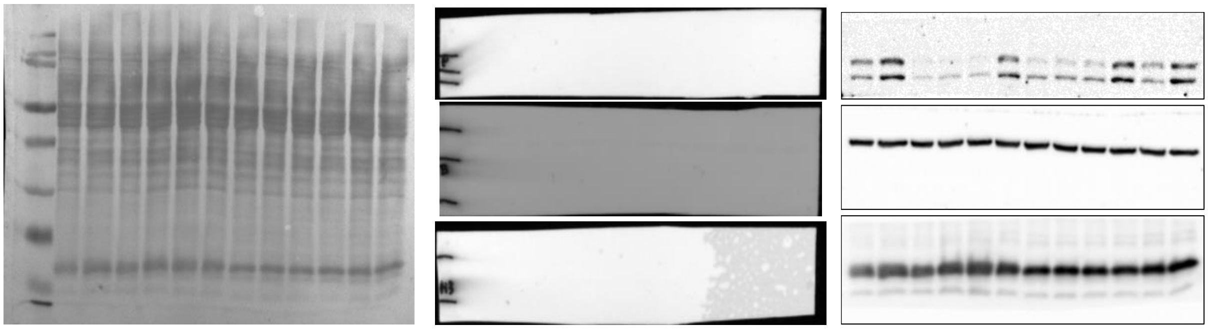

Fig S(a) Blot stained with ponceau S stain.

Fig S(b): White light image of blot. Blot was cut below 25 kDa and below 75 kDa in protein marker and probed with PR(upper portion), β-actin (middle portion) and histone(lower portion) antibody

Fig S(c) : Chemilumiscence image of blot probed with PR(upper portion), β-actin (middle portion) and histone(lower portion)

Supplementary information- V

HPLC profiles of studied compounds

Column Used C18 Mobile Phase Acetonitrile: water (60:40)

SAMPLE INFORMATION

Sample Name:	quercetin	Acquired By:	System
Sample Type:	Unknown	Sample Set Name:	flavones quercetein
Vial:	1	Acq. Method Set:	flavones scientific rport
Injection #:	1	Processing Method:	quercetin hplc
Injection Volume:	10.00 ul	Channel Name:	W2489 ChA
Run Time:	30.0 Minutes	Proc. Chnl. Descr.:	W2489 ChA 272nm
Date Acquired:	8/30/2018 2:53:04 PM IST		
Date Processed:	9/19/2018 5:22:03 PM IST		

Auto-Scaled Chromatogram

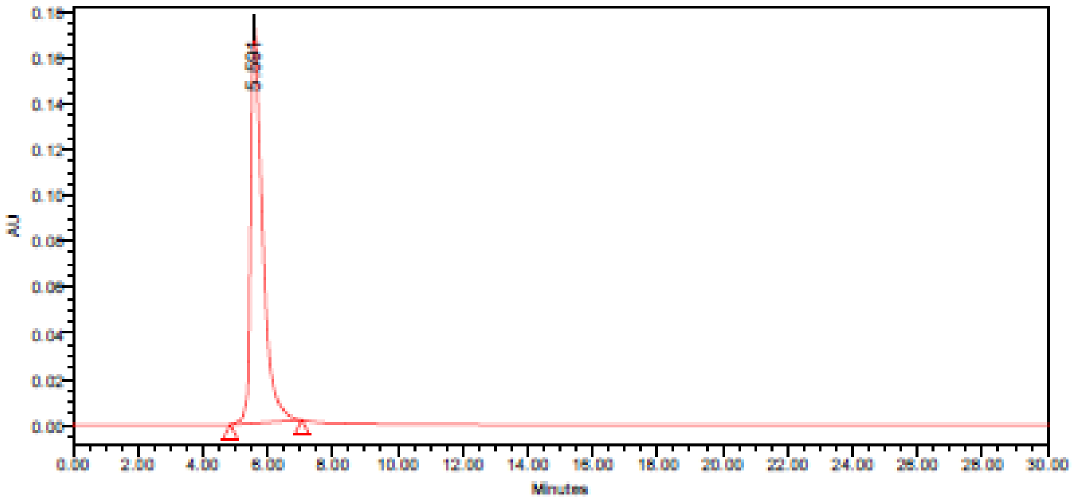

Peak Results

	Name	RT	Area	Height	Amount	Units
1		5.591	4370987	172784		

SAMPLE INFORMATION

Sample Name:	hesperidin	Acquired By:	System
Sample Type:	Unknown	Sample Set Name:	naringin
Vial:	2	Acq. Method Set:	flavones scientific rport
Injection #:	1	Processing Method:	hesperidine hplc
Injection Volume:	10.00 ul	Channel Name:	W2489 ChA
Run Time:	70.0 Minutes	Proc. Chnl. Descr.:	W2489 ChA 280nm
Date Acquired:	9/6/2016 3:42:55 PM IST		
Date Processed:	9/19/2016 5:07:30 PM IST		

Auto-Scaled Chromatogram

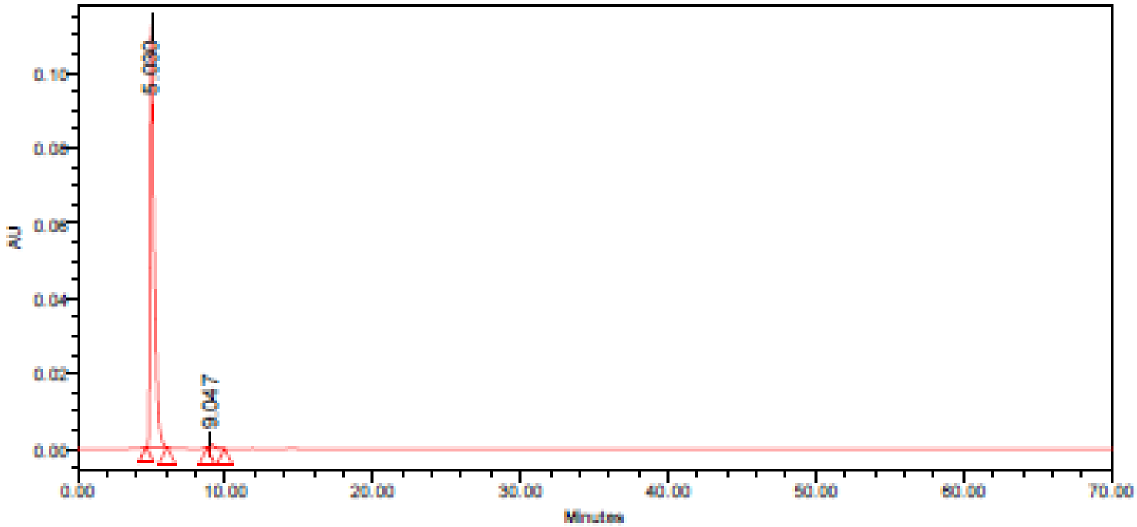

Peak Results

	Name	RT	Area	Height	Amount	Units
1		5.030	2121688	111883		
2		9.047	23864	1013		

SAMPLE INFORMATION

Sample Name:	naringin	Acquired By:	System
Sample Type:	Unknown	Sample Set Name:	naringin
Vial:	1	Acq. Method Set:	flavones scientific rport
Injection #:	1	Processing Method:	naringin hplc
Injection Volume:	10.00 ul	Channel Name:	W2489 ChA
Run Time:	70.0 Minutes	Proc. Chnl. Descr.:	W2489 ChA 280nm
Date Acquired:	9/6/2018 2:31:44 PM IST		
Date Processed:	9/19/2018 5:10:22 PM IST		

Auto-Scaled Chromatogram

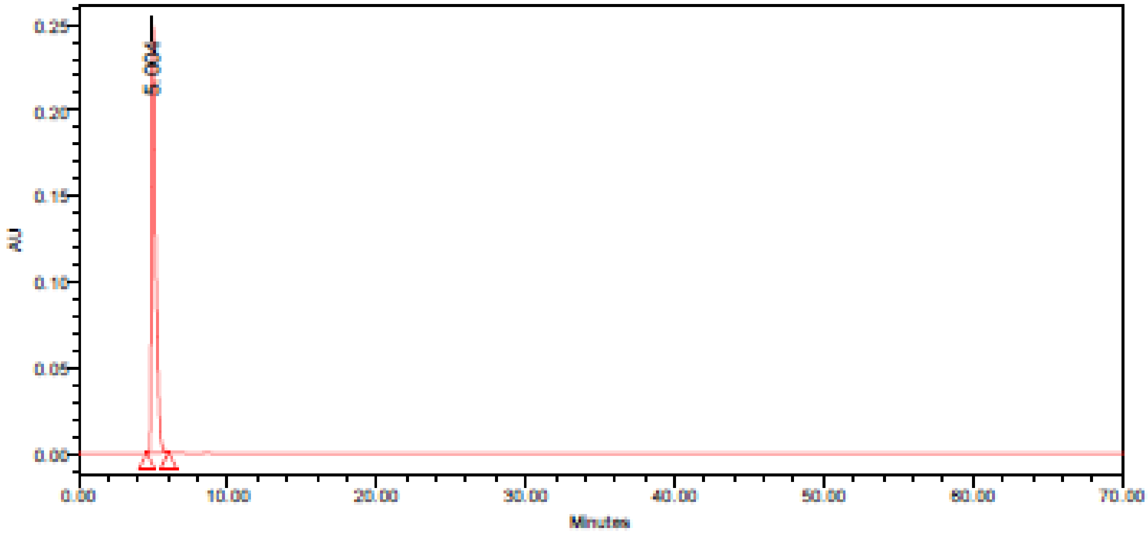

Peak Results

	Name	RT	Area	Height	Amount	Units
1		5.004	4822974	248484		

SAMPLE INFORMATION

Sample Name:	genistin	Acquired By:	System
Sample Type:	Unknown	Sample Set Name:	epicatechin
Vial:	2	Acq. Method Set:	flavones scientific genistein
Injection #:	1	Processing Method:	genistein hplc
Injection Volume:	10.00 ul	Channel Name:	W2489 ChA
Run Time:	60.0 Minutes	Proc. Chnl. Descr.:	W2489 ChA 252nm
Date Acquired:	9/7/2018 12:07:04 PM IST		
Date Processed:	9/19/2018 5:00:20 PM IST		

Auto-Scaled Chromatogram

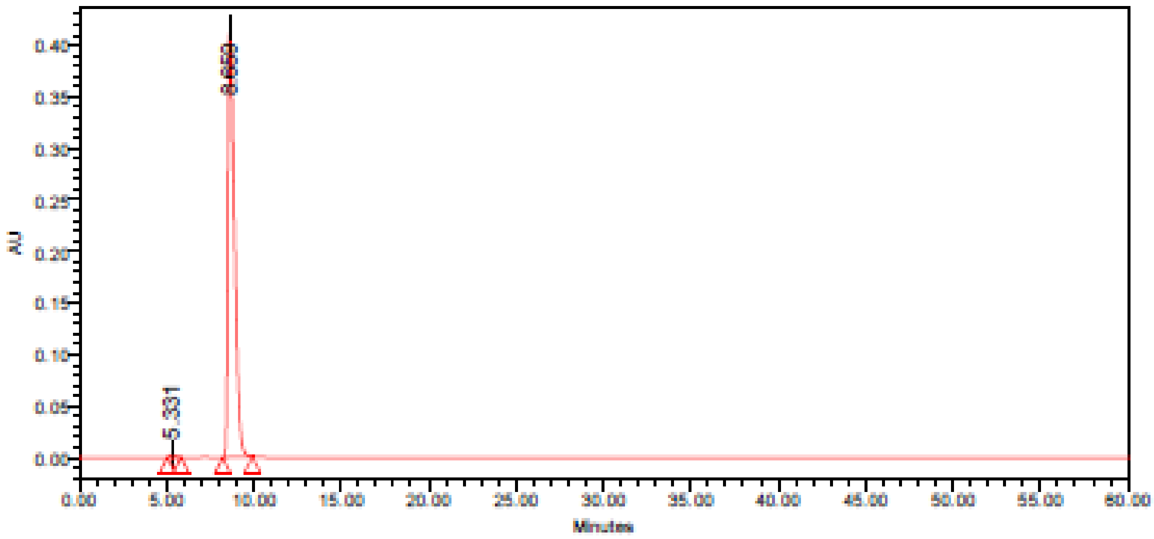

Peak Results

	Name	RT	Area	Height	Amount	Units
1		5.331	58994	2801		
2		8.659	10429641	416890		

SAMPLE INFORMATION

Sample Name:	luteolin	Acquired By:	System
Sample Type:	Unknown	Sample Set Name:	luteolin
Vial:	1	Acq. Method Set:	flavones scientific rport
Injection #:	1	Processing Method:	lutolin hplc
Injection Volume:	10.00 ul	Channel Name:	W2489 ChA
Run Time:	70.0 Minutes	Proc. Chnl. Descr.:	W2489 ChA 250nm
Date Acquired:	9/6/2018 12:02:37 PM IST		
Date Processed:	9/19/2018 5:13:50 PM IST		

Auto-Scaled Chromatogram

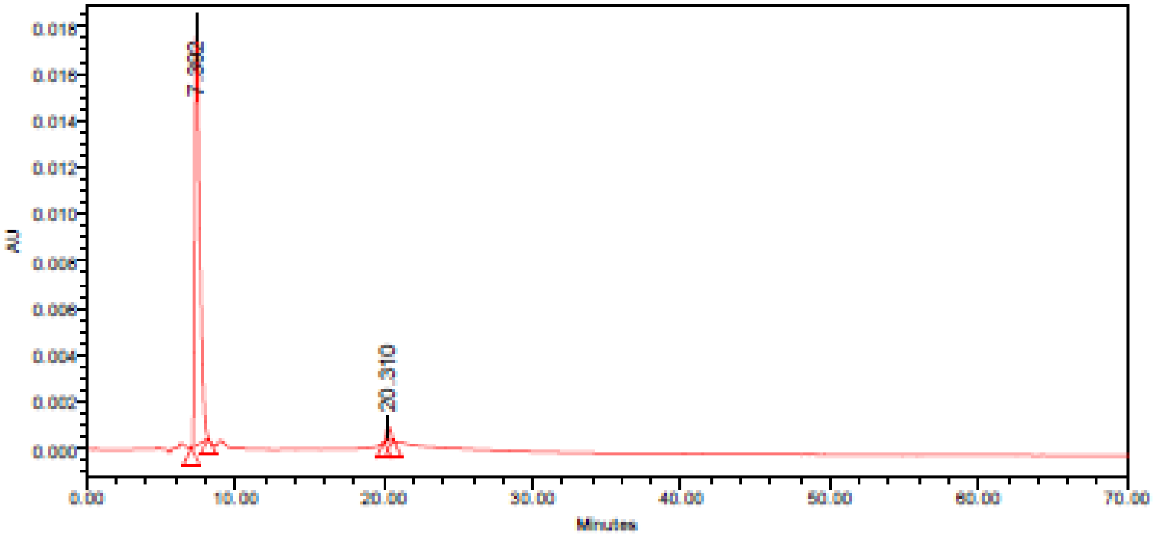

Peak Results

	Name	RT	Area	Height	Amount	Units
1		7.392	412179	17881		
2		20.310	13198	595		

SAMPLE INFORMATION

Sample Name:	galangin	Acquired By:	System
Sample Type:	Unknown	Sample Set Name:	luteolin
Vial:	2	Acq. Method Set:	flavones scientific rport
Injection #:	1	Processing Method:	galangin hplc
Injection Volume:	10.00 ul	Channel Name:	W2489 ChA
Run Time:	70.0 Minutes	Proc. Chnl. Descr.:	W2489 ChA 260nm
Date Acquired:	9/6/2018 1:13:48 PM IST		
Date Processed:	9/19/2018 5:12:12 PM IST		

Auto-Scaled Chromatogram

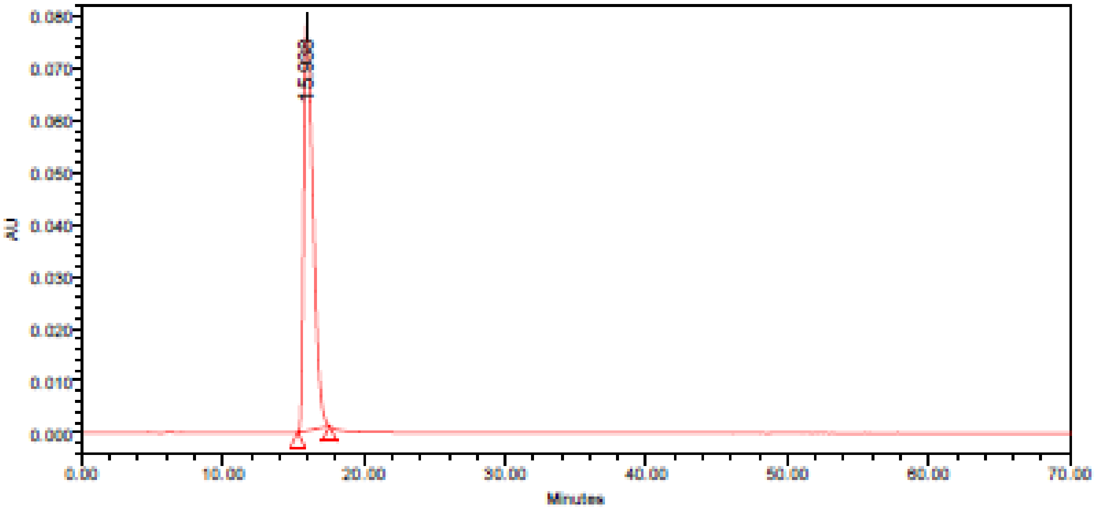

Peak Results

	Name	RT	Area	Height	Amount	Units
1		15.938	3573273	77860		

SAMPLE INFORMATION

Sample Name:	baicalein	Acquired By:	System
Sample Type:	Unknow n	Sample Set Name:	flavones sci rep
Vial:	3	Acq. Method Set:	flavones scientific rport
Injection #:	1	Processing Method:	baicalein hplc
Injection Volume:	10.00 ul	Channel Name:	W2489 ChA
Run Time:	60.0 Minutes	Proc. Chnl. Descr.:	W2489 ChA 272nm
Date Acquired:	9/5/2018 2:38:19 PM IST		
Date Processed:	9/19/2018 5:17:05 PM IST		

Auto-Scaled Chromatogram

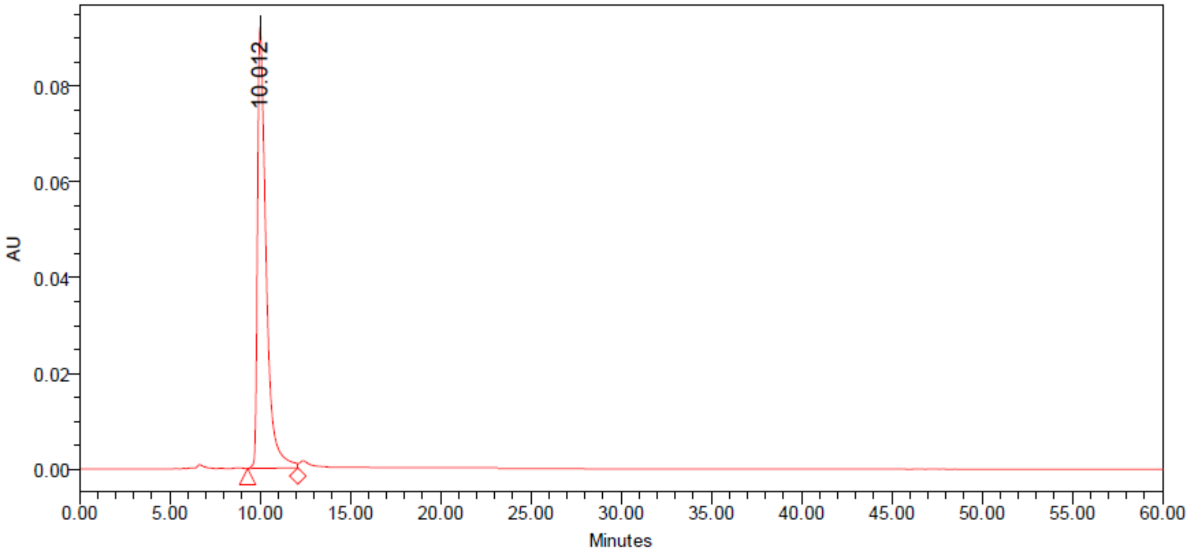

Peak Results

	Name	RT	Area	Height	Amount	Units
1		10.012	3061312	92140		

SAMPLE INFORMATION

Sample Name:	epicatechin	Acquired By:	System
Sample Type:	Unknown	Sample Set Name:	epicatechin
Vial:	1	Acq. Method Set:	flavones scientific rport
Injection #:	1	Processing Method:	epicatechin hplc
Injection Volume:	10.00 ul	Channel Name:	W2489 ChA
Run Time:	60.0 Minutes	Proc. Chnl. Descr.:	W2489 ChA 280nm
Date Acquired:	9/7/2018 1:14:55 PM IST		
Date Processed:	9/19/2018 4:52:11 PM IST		

Auto-Scaled Chromatogram

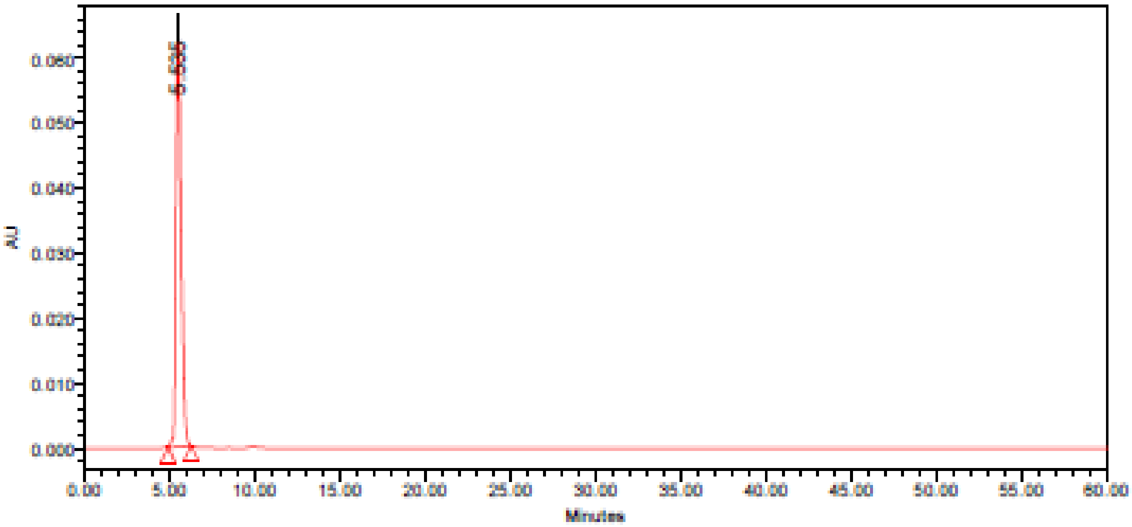

Peak Results

	Name	RT	Area	Height	Amount	Units
1		5.535	1326137	64551		

SAMPLE INFORMATION

Sample Name:	myricitin	Acquired By:	System
Sample Type:	Unknown	Sample Set Name:	flavones sci rep
Vial:	2	Acq. Method Set:	flavones scientific rport
Injection #:	1	Processing Method:	myricitin hplc
Injection Volume:	10.00 ul	Channel Name:	W2489 ChA
Run Time:	70.0 Minutes	Proc. Chnl. Descr.:	W2489 ChA 272nm
Date Acquired:	9/5/2018 1:27:09 PM IST		
Date Processed:	9/19/2018 5:18:51 PM IST		

Auto-Scaled Chromatogram

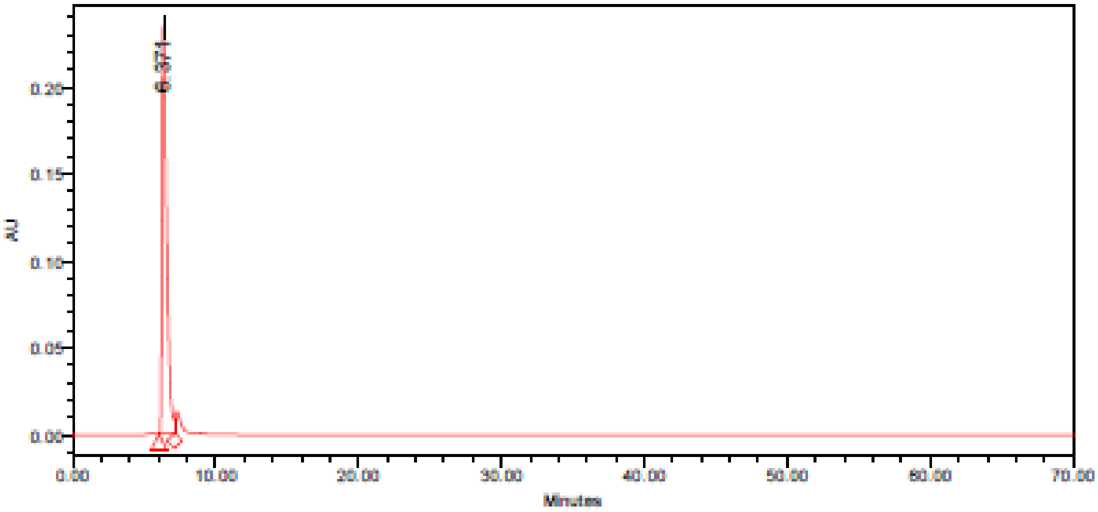

Peak Results

	Name	RT	Area	Height	Amount	Units
1		6.371	5152253	234715		

SAMPLE INFORMATION

Sample Name:	kaempferol	Acquired By:	System
Sample Type:	Unknown	Sample Set Name:	flavones sci rep
Vial:	4	Acq. Method Set:	flavones scientific rport
Injection #:	1	Processing Method:	kaempferol hplc
Injection Volume:	10.00 ul	Channel Name:	W2489 ChA
Run Time:	60.0 Minutes	Proc. Chnl. Descr.:	W2489 ChA 272nm
Date Acquired:	9/5/2018 3:39:30 PM IST		
Date Processed:	9/19/2018 5:15:15 PM IST		

Auto-Scaled Chromatogram

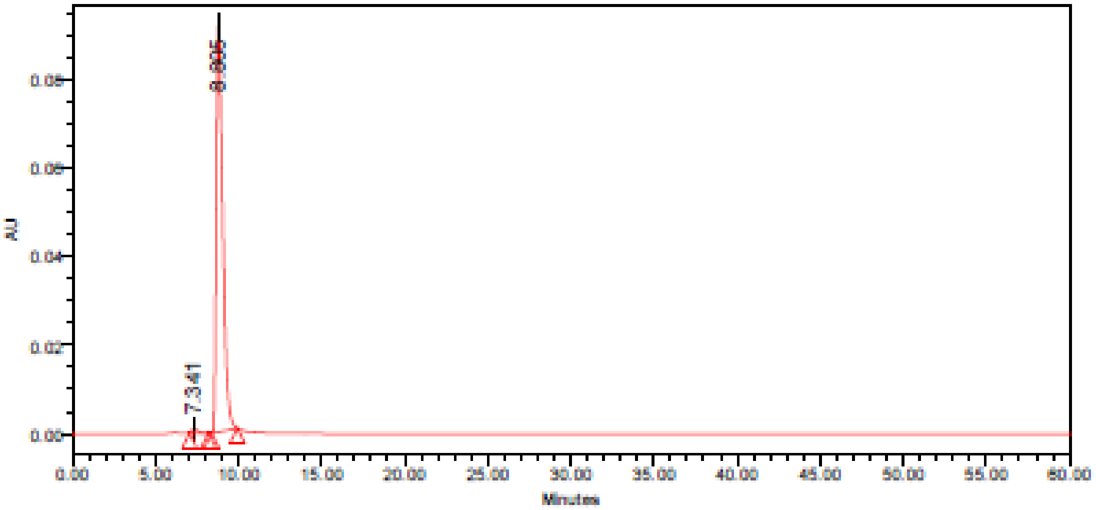

Peak Results

	Name	RT	Area	Height	Amount	Units
1		7.341	24093	1028		
2		8.805	2449949	91651		
